# Transcriptome-wide marker gene expression analysis of stress-responsive sulfate-reducing bacteria

**DOI:** 10.1038/s41598-023-43089-8

**Published:** 2023-09-27

**Authors:** Kalimuthu Jawaharraj, Vincent Peta, Saurabh Sudha Dhiman, Etienne Z. Gnimpieba, Venkataramana Gadhamshetty

**Affiliations:** 1 Civil and Environmental Engineering, South Dakota Mines, 501 E. St. Joseph Street, Rapid City, SD 57701 USA; 2 2D-Materials for Biofilm Engineering, Science and Technology (2D BEST) Center, South Dakota Mines, 501 E. St. Joseph Street, Rapid City, SD 57701 USA; 3 Data-Driven Materials Discovery for Bioengineering Innovation Center, South Dakota Mines, 501 E. St. Joseph Street, Rapid City, SD 57701 USA; 4 Biomedical Engineering, University of South Dakota, 4800 N Career Ave, Sioux Falls, SD 57107 USA; 5 Chemistry, Biology and Health Sciences, South Dakota Mines, 501 E. St. Joseph Street, Rapid City, SD 57701 USA

**Keywords:** Biological techniques, Biotechnology, Computational biology and bioinformatics, Microbiology, Molecular biology

## Abstract

Sulfate-reducing bacteria (SRB) are terminal members of any anaerobic food chain. For example, they critically influence the biogeochemical cycling of carbon, nitrogen, sulfur, and metals (natural environment) as well as the corrosion of civil infrastructure (built environment). The United States alone spends nearly $4 billion to address the biocorrosion challenges of SRB. It is important to analyze the genetic mechanisms of these organisms under environmental stresses. The current study uses complementary methodologies, viz*.,* transcriptome-wide marker gene panel mapping and gene clustering analysis to decipher the stress mechanisms in four SRB. Here, the accessible RNA-sequencing data from the public domains were mined to identify the key transcriptional signatures. Crucial transcriptional candidate genes of *Desulfovibrio* spp. were accomplished and validated the gene cluster prediction. In addition, the unique transcriptional signatures of *Oleidesulfovibrio alaskensis* (OA-G20) at graphene and copper interfaces were discussed using in-house RNA-sequencing data. Furthermore, the comparative genomic analysis revealed 12,821 genes with translation, among which 10,178 genes were in homolog families and 2643 genes were in singleton families were observed among the 4 genomes studied. The current study paves a path for developing predictive deep learning tools for interpretable and mechanistic learning analysis of the SRB gene regulation.

## Introduction

Sulfate-reducing bacteria (SRB) are the most widely studied bacteria that cause severe MIC of metals including low carbon steel, copper and nickel, etc.,^1^. Microbial community research revealed that *Desulfovibrio* sp. is the predominant SRB in various MIC sites including internal rust layers on carbon steel^2^, steel pipe transporting oily seawater^3^, metallic surfaces exposed to oil field produced waters^4^ and rust layers on metal plates submerged in seawater^5^. Several genetic mechanisms have been proposed that demonstrate the microbial interactions of SRB with metals, but they are equally exclusive which requires painstaking examination^1^.

Recently, corrosion-resistant coatings on metals using graphene (Gr), hexagonal boron nitride (hBN), and sulfur-selenium (S-Se) alloys were reported^6–8^. Furthermore, the design of effective anti-corrosive coatings is evident if the functional genes and their molecular mechanisms are studied thoroughly^9^. A wide array of analytical techniques *e.g.*, microarray^10^, semiquantitative-PCR^11^, real-time PCR^12^, and high-throughput transcriptomics^13^ are reported for monitoring the SRBs gene expression profiling. As a result, significant variations are observed in the gene datasets displaying minimal overlaps mainly due to the different techniques employed^14,15^.

Datasets from the genome of OA-G20, previously *Desulfovibrio alaskensis* or *Desulfovibrio desulfuricans* have been widely used to mine the essential gene sets and their biological networks that laid the foundation of genome-wide investigation using machine learning approaches^16^. Transcription processes are regulated by several factors and in this complex process the spatiotemporal patterns are attained through the combinatorial function of the genes in regulatory networks^17^. To behave coordinately, the subsets of genes will possess the right combination of regulators while the other genes follow expression patterns that are independent of the culture conditions^18^. The molecular processes are regulated by complicated transcriptional regulatory networks (TRNs) which possess inherent knowledge and frameworks of biological and developmental processes^19^. These TRNs are essential components that explain the interrelationships between adaptive stress response and defense/repair mechanism.

Gene clustering by protein–protein interactions (PPI) is used to extract the cohorts of genes that are co-expressed under a specific experimental condition among the complete set of genes^20,21^. Because of the complexity of the biological networks and the large number of gene sets, several gene clustering algorithms were developed for transcriptomic data^22^. Our previous studies revealed a complex anomalous behavior of Gr, hBN, and alloy coatings on metals along with differential genotypes and phenotypic responses in OA-G20 upon exposure to metals^6–8^. Also, the lower lattice size (~ 0.3 nm) of Gr and hBN is smaller than the sizes of bacteria and their metabolites, and thus they play a role as an ion-permeable membrane barrier^23,24^. These protective coatings offer two major advantages thereby protecting the corroding bacteria from copper toxicity and the underlying metallic substrate from MIC. Furthermore, due to the lack of deep understanding of these interactions at the protein level, both in-vitro and in-silico studies were limited. Our knowledge of proteins crucial for MIC with various metals is still very limited at best. Many of these proteins do not have well-defined structures, let alone functions, making this task of studying MIC-associated proteins even more difficult. Being able to study protein structures and metal surface interactions could also help us to understand how these bacteria first adhere to metal surfaces and start the biofilm formation process and continue with the corrosion of a specific metal. Thus, we hypothesize that comprehensive information on the candidate gene/ proteins involved in the stress responses of the SRBs could benefit in approaching the suitable anti-corrosive coatings to prevent the various stages of biofilm including biofilm conditioning, growth, and biofilm maturation of SRBs. The central goal of this study was to analyze and compare the transcriptome- based gene clusters of in-house generated data and publicly available gene expression datasets of crucial SRBs from Gene Expression Omnibus. The graphical abstract illustrates the schematic of the overall steps and bioinformatic pipelines employed in this study. Here, the OA-G20 cultures were exposed to polycrystalline pristine copper (P-Cu) and single-layered graphene-coated polycrystalline copper (SLG-Cu) and their transcriptome were compared against the controls. The differential transcriptome of SRBs (RNA-seq data from the public domain) exposed to three different experimental stresses was also evaluated. Here, a subset of genes and proteins involved in OA-G20-copper-graphene interactions that could play a crucial role in stress handling and the subsequent phenotypic responses were identified. In addition, the differential transcriptome of *Desulfovibrio* spp. based on gene expression analysis, gene ontology (GO) enrichment, and gene clustering analysis were also compared and evaluated.

## Results and discussion

### Differential gene expression (DGE) analysis in OA-G20

**Figure 1:**
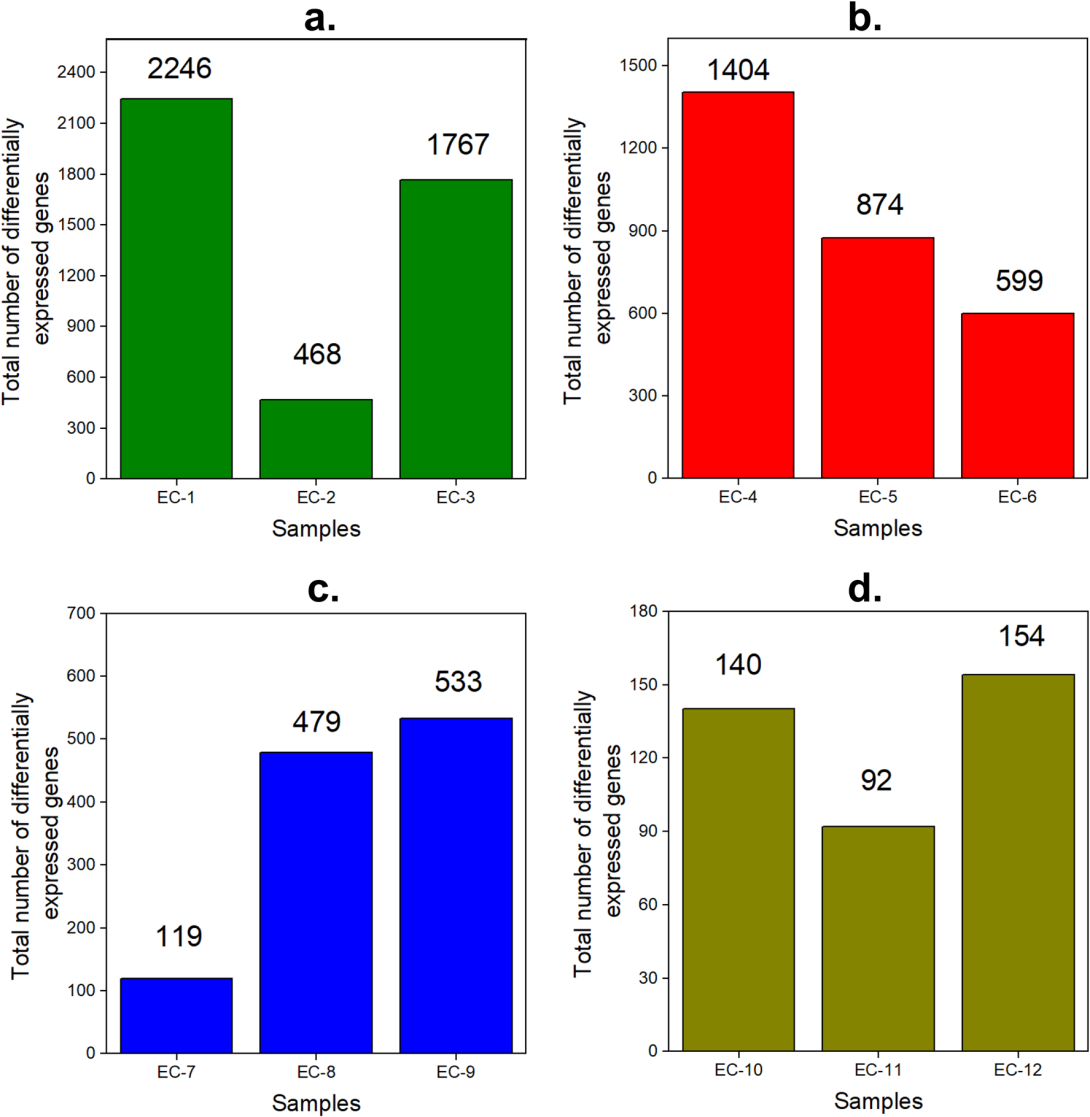
Total number of DEGs of the RNA-seq datasets evaluated in this study. (**a**) OA-G20 exposed to copper stress (**b**) *M. hydrothermalis* exposed to hydrostatic stress (**c**) *P. piezophilus* exposed to hydrostatic stress and (**d**) *D. vulgaris* exposed to CuO stress. EC-1 to EC-12 corresponds to 12 different experimental stresses. See Table 1 for detailed explanations.

The cDNA libraries of the planktonic cultures were constructed, sequenced, and created with a total of 18 M to 23 M reads generated for the three replicates which were then mapped onto the reference genome of OA-G20. In this study, the RNA-seq analysis revealed the number of differentially expressed genes among the three different experimental conditions. This analysis revealed both the up-regulated and down-regulated genes under the above-mentioned experimental conditions. The DGE analysis after normalization showed 2246, 468, and 1767 genes (Fig. 1a) were statistically significant DGE in EC-1, EC-2, and EC-3, respectively for 7 days of incubation. More genes were differentially expressed in EC-1 and EC-3 compared to EC-2. Results also showed that 69%, 37%, and 46%, were up-regulated in EC-1, EC-2, and EC-3, respectively. Whereas 31%, 63%, and 54% were down-regulated in EC-1, EC-2, and EC-3, respectively. The most highly up-regulated and down-regulated genes in EC-1 are oligopeptide transporter OPT superfamily protein (7.8-folds) and phage P22, anti-repressor protein (-9.11-folds), respectively. This indicates the metabolism towards membrane transport of oligopeptides is elevated in control conditions. Whereas the phage P22, anti-repressor protein functions of OA-G20 have not been reported so far. The most highly up-regulated and down-regulated genes in EC-2 are glycosyl transferase family 2 (8.5 folds) and tripartite ATP-independent periplasmic transporter DctQ component (-5.3-folds), respectively. This shows that the transferase family proteins are elevated when exposed to P-Cu. Glycosyltransferases constitute a family of proteins that are involved in the biosynthesis of disaccharide, oligosaccharides, and polysaccharides that performs a wide range of functions from structural features and storage to signaling^25^. The induction of polysaccharide biosynthesis could be attributable to a stress response trait of sulfate-reducing bacteria^26^. The most highly up-regulated and down-regulated genes in EC-3 are DNA binding domain protein, excisionase family (8.3-folds), and Glycosyl transferase family 2 (-7-folds), respectively. This indicates that the molecular functions regarding the DNA binding and catalytic activities were up-regulated during EC-3. The DNA-binding protein, excisionase family is a phage-encoded excisionase domain as a transcriptional regulator mainly involved in excisive recombination in regulating the intasome assembly and inhibits viral integration^27^. On another note, the maximal down-regulation of glycosyl transferase family 2 proteins depicts that the graphene coating could be inhibiting the stress-responsive genes related to saccharide biosynthesis compared to P-Cu. The transcriptome-wide analysis of bacterial-graphene interactions is scarce. The biofunctionalization of graphene oxide-copper nanocomposite was reported to disengage the cariogenic *Streptococcus mutans* optimal biofilm thereby disrupting the exopolysaccharide matrix biosynthesis and dysregulation of exopolysaccharide and biofilm-associated genes^28^. Furthermore, the functionalized graphene oxide on cellulose fibers inhibited the growth of the methicillin-resistant *Staphylococcus aureus* (MRSA) strain. This also triggered their transcriptome with regulatory changes in genes related to biofilm virulence and the arginine metabolism. Also, the two-component systems were repressed whereas the siderophore biosynthetic genes were induced^29^. The heat map analysis revealed the DGE pattern of selected 279 genes from EC-1, EC-2, and EC-3 (Fig. 3a.). The color in the heatmap represents the log2FC and the extra column next to the dendrograms represents the unique genes. The number in this column shows the clusters of genes formed based on the dendrograms. It was observed that 12 gene clusters were formed among the corresponding experimental conditions.

**Table 1:** RNA-seq data collection: The major characteristics of each BioProjects evaluated in this study, along with their library layout, RNA-seq platform, and the SRA numbers used for DGE analysis.

S. no	SRB strain (total number of genes)	BioProject Number	Type of stress	SRA/ Geo accession	Layout	References
1	*Oleidesulfovibrio alaskensis* (3350)	PRJNA1003487	Copper stress	GSE240798	Illumina	This study
	EC-1 (control VS P-Cu)					
	EC-2 (P-Cu VS SLG-Cu)					
	EC-3 – (SLG-Cu VS control)					
2	*Maridesulfovibrio hydrothermalis* (3330)	PRJNA240876	Hydrostatic stress	GSE55745	Illumina	^33^
	EC-4 (0.1 MPa VS 10 MPa)					
	EC-5 (10 MPa VS 26 MPa)					
	EC-6 (26 MPa VS control)					
3	*Pseudodesulfovibrio piezophilus* (3329)	PRJNA251861	Hydrostatic stress	GSE58269	High throughput	^34^
	EC-7 (0.1 MPa VS 10 MPa)					
	EC-8 (10 MPa VS 26 MPa)					
	EC-9 (26 MPa VS control)					
4	*Desulfovibrio vulgaris* (3172)	PRJNA395924	CuO nanoparticles	GSE101911	Illumina	^35^
	EC-10 (control VS 25 mg/L CuO)					
	EC-11 (25 mg/L CuO VS 250 mg/L)					
	EC-12 (250 mg/L CuO VS control)					
5	*Desulfovibrio vulgaris* (3172)	PRJNA314088	Free nitrous acid	GSE78834	Illumina	^36^
	EC-13 (Control VS 4 μg N/L)					

### Influences of P-Cu and SLG-Cu on the transcriptional sketches

#### Regulation of glycosyltransferase family proteins

Four different RNA-seq datasets of SRBs were finalized and evaluated for further analysis (Table 1). The datasets GSE58269 (*Pseudodesulfovibrio piezophilus*) and GSE55745 (*Maridesulfovibrio hydrothermalis*) have duplicates with three experimental conditions. Where the dataset GSE78834 (*Desulfovibrio vulgaris*) had triplicates of the gene expression profiles with two experimental conditions. And the dataset GSE101911 (*Desulfovibrio vulgaris*) does not have any replicates with experimental conditions. Thus, there were 8 control and 13 test samples (subjected to stress) that were available for further analysis. All sample codes, number of genes, BioProject number, SRA, GEO accession numbers and their experimental conditions are provided in Table.1. To identify the candidate genes involved in overall genetic regulation after P-Cu and SLG-Cu exposure, the top ten up and down-regulated genes under EC-1, EC-2, and EC-3 (Tables 2, 3 and 4) were chosen for further genetic analysis. Interestingly, the glycosyl transferase group 1 protein (Dde_0593) was up-regulated in control (6.7-folds), the same protein was down-regulated in P-Cu (-4.7-folds), and SLG-Cu (-1.5-folds), respectively. On the other hand, the glycosyl transferase family 2 protein (Dde_2876) showed a counterintuitive expression profile of up-regulation in P-Cu (8.5-folds) and down-regulation in SLG-Cu (-7-folds), respectively. But this indicates that the glycosyl transferase pathway (Family 1 and 2 proteins) is critically down-regulated under graphene coatings. Interestingly, mutations in glycosyltransferase proteins of OA-G20 revealed reduced biofilm formation suggesting that these proteins are directly involved in cell-to-cell interactions^30^. Bacterial glycosyltransferases are mainly involved in the biosynthesis and transferring of the nucleotide sugar precursors to the sugar/ non-sugar acceptors resulting in the production of exopolysaccharides, lipopolysaccharides, glycolipids, and peptidoglycans^25,31,32^.

**Table 2:** Top ten up and down-regulated genes with their gene IDs, log2 FC (fold change) values, standard errors (Std Error), and the corresponding protein names in EC-1.

Gene ID	Log2 FC	Std error	Protein name
Down (EC-1)			
Dde_1739	− 9.11	1.18	Phage P22, antirepressor protein
Dde_4057	− 7.22	0.14	DNA binding domain protein, excisionase family
Dde_0715	− 6.72	0.13	Uncharacterized protein
Dde_3197	− 6.55	0.10	Uncharacterized protein
Dde_2670	− 6.01	0.13	Ferrous iron transporter component feoA
Dde_3199	− 5.98	0.12	Uncharacterized protein
Dde_3667	− 5.83	0.11	Flavodoxin
Dde_3198	− 5.79	0.18	Dinitrogenase iron-molybdenum cofactor biosynthesis protein
Dde_0979	− 5.74	0.14	Uncharacterized protein
Dde_3226	− 5.60	0.09	Phage shock protein A, PspA
Up (EC-1)			
Dde_0728	7.79	1.06	Oligopeptide transporter OPT superfamily protein
Dde_2556	7.14	1.06	Phage uncharacterized protein
Dde_3671	7.01	1.06	ABC-type transporter, integral membrane subunit
Dde_0547	6.97	1.06	ComEC/Rec2-related protein
Dde_3106	6.86	1.07	ABC-type transporter, periplasmic subunit
Dde_3661	6.75	1.06	Phospholipid/glycerol acyltransferase
Dde_0635	6.71	1.07	Tripartite ATP-independent periplasmic transporter DctQ component
Dde_0593	6.68	1.07	Glycosyl transferase group 1
Dde_2829	6.43	1.06	Major facilitator superfamily MFS_1
Dde_0244	6.33	1.06	PP-loop domain protein

**Table 3:** Top ten up and down-regulated genes with their gene IDs, log2 FC values, standard errors, and the corresponding protein names in EC-2.

Gene ID	Log2 FC	Std error	Protein name
Down (EC-2)			
Dde_0635	− 5.33	1.29	Tripartite ATP-independent periplasmic transporter DctQ component
Dde_0547	− 5.28	1.31	ComEC/Rec2-related protein
Dde_2384	− 5.23	1.30	Response regulator receiver
Dde_0728	− 5.18	1.31	Oligopeptide transporter OPT superfamily protein
Dde_2493	− 4.98	1.33	Type III restriction protein res subunit
Dde_2362	− 4.89	1.33	Response regulator receiver protein
Dde_1959	− 4.84	1.34	Selenium metabolism protein YedF
Dde_1552	− 4.82	1.35	HTH-type transcriptional regulatory protein TyrR
Dde_1551	− 4.77	1.34	D-alanyl-D-alanine dipeptidase (D-Ala-D-Ala dipeptidase)
Dde_0593	− 4.69	1.35	Glycosyl transferase group 1
Up (EC-2)			
Dde_2876	8.46	1.57	Glycosyl transferase family 2
Dde_2791	5.82	1.36	Arsenical-resistance protein
Dde_3493	5.73	1.27	Nitrogen regulatory protein P-II
Dde_2819	4.23	1.02	Uncharacterized protein
Dde_2375	3.66	1.47	Heavy metal-binding domain-containing protein
Dde_2235	3.48	1.62	50S ribosomal protein L36
Dde_3449	3.45	1.68	4Fe-4S ferredoxin-type domain-containing protein
Dde_3226	3.37	0.21	Phage shock protein A, PspA
Dde_0814	3.32	1.66	Uncharacterized protein
Dde_3071	3.21	1.50	Polar amino acid ABC transporter, inner membrane subunit

**Table 4:** Top ten up and down-regulated genes with their gene IDs, log2 FC values, standard errors, and the corresponding protein names in EC-3.

Gene ID	Log2 FC	Std error	Protein name
Down (EC-3)			
Dde_2876	− 6.99	0.32	Glycosyl transferase family 2
Dde_3617	− 4.48	0.92	Anthranilate synthase
Dde_3105	− 4.26	0.99	Iron-chelate-transporting ATPase
Dde_1195	− 4.24	0.99	Nitroreductase
Dde_0284	− 4.24	0.93	Uncharacterized protein
Dde_2456	− 4.10	0.99	PAS domain containing protein
Dde_3493	− 4.05	0.99	Nitrogen regulatory protein P-II
Dde_0508	− 4.05	0.99	Signal peptide peptidase SppA, 36 K type
Dde_2791	− 3.97	0.46	Arsenical-resistance protein
Dde_3485	− 3.88	0.94	Prephenate dehydrogenase
Up (EC-3)			
Dde_4057	8.35	0.11	DNA binding domain protein, excisionase family
Dde_3199	7.67	0.09	Uncharacterized protein
Dde_3198	7.49	0.15	Dinitrogenase iron-molybdenum cofactor biosynthesis protein
Dde_4005	6.90	0.15	Uncharacterized protein
Dde_2670	6.80	0.09	Ferrous iron transporter component feoA
Dde_1739	6.79	1.24	Phage P22, antirepressor protein
Dde_3197	6.76	0.15	Uncharacterized protein
Dde_3667	6.72	0.08	Flavodoxin
Dde_0715	6.28	0.24	Uncharacterized protein
Dde_3413	5.96	0.09	ATPase AAA-2 domain protein

#### Unique nitrogen metabolism in OA-G20

It is also noteworthy to mention that dinitrogenase iron-molybdenum cofactor biosynthesis protein (Dde_3198) was down-regulated in control (-5.8-folds) and P-Cu (-1.3-folds), respectively. Interestingly, the Dde_3198 protein was found to be strongly up-regulated (7.5-folds) in SLG-Cu. This Dde_3198 protein codes for the iron-molybdenum co-factor biosynthesis in the dinitrogenase enzyme of nitrogenase enzyme complex of nitrogen-fixing bacteria and is involved in dinitrogen reduction to ammonia^37^. The protein sequence homology analysis of Dde_3198 using UniProt revealed that this protein of OA-G20 showed sequence homology with several dinitrogenase iron-molybdenum cofactor biosynthesis protein of phylogenetically related *Desulfovibrio* sp. This included *Desulfovibrio psychrotolerans* (67.2%), *Desulfobaculum xiamenense* (63%), *Desulfohalobium retbaense* (59.7%), *Desulfocurvibacter africanus* (58.8%), *Desulfovibrio sulfodismutans* (58.8%) and *Desulfovibrio ferrophilus* (58%). In addition, the Dde_3197 gene showed a maximum homology of 41.3% with dinitrogenase iron-molybdenum cofactor biosynthesis protein of *Maridesulfovibrio salexigens*. Also, nitrogen fixation and associated gene clusters have been well-reported in sulfate-reducing bacterial pure cultures and sediment consortia^38,39^. In addition, the nitrogen regulatory protein P-II (Dde_3493) was slightly down-regulated in control (-1.2-folds) and SLG-Cu (-4folds), respectively, whereas a significant up-regulation was observed in P-Cu (5.7 folds). Although the nitrogen fixation pathways of OA-G20 were not completely understood, the current study depicts that the nitrogen fixation pathway is activated upon the graphene exposure. This result corroborates the previous report on the enhanced nitrogen fixation and growth of *Azotobacter chroococcum* by reduced graphene oxide^40^. This finding highlights the stimulating effects of graphene on nitrogen fixation and the eco-friendly aspects of graphene with minimal bactericidal properties.

#### Up-regulation of translation pathway

Bacteria respond rapidly to environmental stimuli through translation machinery by fine-tuning their specific protein levels using programmed ribosome-inducing or pausing frameshifting^41^. In this study, predominant genes involved in translation machinery were down-regulated in control (46 down-regulated and 7 up-regulated). Interestingly, the translation-related genes were up-regulated on both P-Cu and SLG-Cu. This confirms that the P-Cu (16 up-regulated and 2 down-regulated) and SLG-Cu (46 up-regulated and 6 down-regulated) exposure is activating the translation mechanism by activating suitable proteins to withstand the environmental stimuli. This result is contradictory to the previous report on OA-G20 exposed to dissolved copper ions that revealed complete down-regulation of the translation machinery^13^.

### Transcriptional signatures related to stress responses

Interestingly, our previous studies revealed the unique aggressive response of SLG-Cu exposed to OA-G20 showing a fivefold higher biogenic sulfide attack than P-Cu. In contrast, the multi-layered graphene on Cu (MLG-Cu) coatings on P-Cu restricted the attack of OA-G20 by tenfold and 1.4-folds than SLG-Cu and P-Cu, respectively^7^. Also, we observed that OA-G20 attains the stationary phase growth condition upon prolonged exposure of 7–8 days of incubation and forms dense biofilm matrix showing unique signatures of rod-shaped OA-G20 cells and matured biofilm microstructures. This ensures that the biofilm was mature enough to take the planktonic cells for further omics analysis. This concept also corroborates with the previous reports of biofilm-like behavior of planktonic cells of *Staphylococcus epidermidis*^42^. Interestingly, most of the differential protein expressions of two strains of *S. epidermis* were observed in planktonic cultures than the sessile counterparts. Especially, a remarkable higher expression of candidate stress response proteins including putative universal proteins and S-ribosylhomocysteine lyase, a regulator of the quorum sensing (QS) revealed the harsh culture conditions of planktonic *S. epidermis*. Similarly, another oxidative stress responsive cytoplasmic protein like hydroperoxide resistance cytoplasmic protein were under expressed in sessile cells than planktonic counterparts. The major reasons could include the low oxygen levels, nutrient shortage and bulk accumulation of biofilm cells and catabolites makes the sessile cells inaccessible to the metabolites and the biofilm microstructures. Thus, genetic interaction between the planktonic and biofilm cells are the crucial communication key for the “*Living Together*” in biofilms. Also, this provides comprehensive details on the alternating transition cycles of bacterial biofilms to switch in two ways such as (i). from planktonic to biofilm (ii) from biofilm to detached, newly planktonic cells. This hypothesis on the biofilm-like behavior of planktonic cells were further corroborated by the cytological and CLSM analyses showing the vigorous stressful growth conditions of *Staphylococci* resulted in the aggregation of free-floating cells and remarkable metabolic activities to withstand stress than the sessile counterparts^42,43^. In another study, the RT-PCR-based gene expression analysis in planktonic OA-G20 revealed that multi-layered hexagonal boron nitride (ML-hBN) and few-layer hexagonal boron nitride (FL-hBN) triggered a 2.7-folds and 1.6-folds up-regulation of *tadC* (Flp pilus assembly protein) compared to P-Cu, respectively. These *tadC* proteins could have promoted the cell-to-cell interactions and EPS biosynthesis that favored thicker biofilms and eventually enhanced biogenic sulfide attacks than control^8^. We also reported the antibacterial behavior of sulfur-selenium (S-Se) coatings on low-carbon steel exposed to OA-G20, which reveals the potency of S-Se coatings on biofilm suppression and eventually MIC mitigation. Thus, considering the recent evidence on the biofilm-like phenotypes of the free-floating cells and the highly biodynamic biofilm biology this present study was designed to exclusively unravel the unique planktonic transcriptional anomalies of OA-G20 exposed to P-Cu and SLG-Cu. The genes were segregated based on their GO term IDs and classified into six crucial functions that are related to electrochemical interactions and biofilms. This includes cell motility/ flagella, riboflavin biosynthesis/ FMN binding proteins, electron transfer activity, lactate oxidation, and sulfate reduction, quorum sensing, two-component systems/EPS-related genes, and stress response genes (Supplementary Table S1). In addition, the top six enriched GOs and their total gene count in EC-1, EC-2, and EC-3 were tabulated in Supplementary Table S2A–C, respectively.

#### Electron transfer activity

Bacterial electron transfer activity is a molecular entity that can serve as an electron donor and electron acceptor in an electron transport chain to generate a transmembrane electrochemical gradient^44^. This study showed more up-regulation of electron transfer genes in control (P-Cu) with a z-score of 0.11 whereas the SLG-Cu with a z-score of 0 revealed equal up and down-regulation tendencies among the candidate genes on electron transfer activity. The major proteins involved in the electron transport chains were cytoplasmic ferredoxin, non-heme iron-containing protein rubredoxin, tetraheme cytochrome c3, and flavoprotein flavodoxin^45^. Interestingly, the up-regulated genes in EC-3 were rubredoxin (Dde_3194), periplasmic (Fe) hydrogenase small subunit (Dde_0082), aldehyde ferredoxin oxidoreductase (Dde_2460), and formate dehydrogenase, alpha subunit (Dde_0717). The rubredoxin proteins are involved in the defense mechanisms against the oxidative stress and electron transfer of *Desulfovibrio* sp.^46^. Whereas the aldehyde ferredoxin oxidoreductase enzymes are involved in the aldehyde catabolism and could be used by confurcating hydrogenase to form hydrogen which is used during sulfate reduction^47,48^. In addition, the periplasmic hydrogenase of *Desulfovibrio* sp. mainly facilitates the oxidation of molecular hydrogen^49^.

#### Riboflavin biosynthesis/ FMN binding proteins

The extracellular electron transfer in SRBs occurs through two different biological mechanisms i.e., direct electron transfer (DET) and mediated electron transfer (MET). This electron transfer can occur between the microbial planktonic cultures/ sessile cultures and the coupons tested. MET occurs through riboflavin (vitamin B2), a second member of the vitamin B complex that is present in a free state, bounded within the extracellular cytochrome/ flavocytochrome, and acts as a redox mediator^50^. Whereas the DET occurs using the cytochromes, pilus, or nanowires^51^. Interestingly, the z-scores of riboflavin biosynthesis/ FMN binding proteins were almost the same in EC-1 and EC-3 were 0.06 (11) and 0.05 (8), respectively. This confirms that the riboflavin biosynthesis and FMN binding proteins in EC-1 (Control) and EC-3 (SLG-Cu) were almost the same. Riboflavin and flavin adenine dinucleotide (FAD) were reported to be the common electron mediators that efficiently enhance the electron transfer by accelerating the pitting corrosion in *D. vulgaris*^52,53^. In OA-G20, riboflavin is an intermediate electron mediator flavin-like endogenously produced protein during the FAD/FMN biosynthesis during enhanced extracellular electron transfer (EET) and biocorrosion^54^.

#### Cell motility and flagella

The bacterial motility using flagella-driven locomotion is directly linked with the bacterial response to environmental stress and chemotaxis^55^. The flagellar mechanism harbors several gene sets in OA-G20, and those crucial GO terms with their z-scores involved in EC-1 and EC-3 were listed in Supplementary Table S1. Results showed that 13 genes were differentially expressed in EC-1 and EC-3. Interestingly, the z-scores of EC-1 and EC-3 were -0.23 and 0.23, respectively. This confirms that the flagellar genetic responses were suppressed and activated in EC-1 (Control) and EC-3 (SLG-Cu), respectively. Flagellar proteins are a complex apparatus that traverses the cell wall that connects the basal body to the whip-like flagellar filament which protrudes outside the bacterial cells^56^. Also, flagella play a major role in transitioning from motile cells to biofilm cells upon exposure to any environmental stimuli in two major steps i.e. inhibition of flagellar rotation and modulation of the basal flagellar reversal frequency^57^. Thus, the up-regulation of the flagellar pathway genes in SLG-Cu denotes enhanced microbial motility as the copper and graphene response.

#### Lactate oxidation

Lactate oxidation is a key metabolic pathway in OA-G20 where the lactate is oxidized by enzymes lactate dehydrogenase and pyruvate-ferredoxin oxidoreductase which then catalyzes pyruvate to acetyl-CoA^58^. Also, approximately 95% of the lactate oxidized by OA-G20 is used for energy generation with the remaining for producing cell materials^59^. In this study, the GO term related to lactate oxidation (Dde_3245, Dde_1843) was down-regulated in EC-2 (P-Cu) whereas the same was up-regulated in SLG-Cu (EC-3) with z-scores -0.04 and 0.04, respectively. This confirms that the lactate oxidation pathway was activated in EC-3 and the graphene coatings on Cu are favoring the same. However, this observation was contradictory to a similar study with down-regulation of lactate oxidation in 15 µM Cu (II) supplementation^13^. This could be due to the lethal concentrations of the copper which might be affecting the lactate oxidation and eventually the electron transport chain.

#### Quorum sensing, two-component systems, and EPS-related genes

During OA-G20 bacterial cell irreversible attachment to metal surfaces, these microbes release cell-signaling molecules including autoinducers (acyl homoserine lactones, AHL) through the quorum sensing (QS) mechanism^60^. As soon as the bacterial cell density reaches a threshold, the QS cell-to-cell communication system releases the autoinducer molecules that trigger a cascade of reactions to form conditioning film proteins, a special class of attachment proteins. Eventually, these cells produce EPS and form intact biofilm onto the attached surface which allows electrochemical interactions at the metal-OA-G20 interface leading to biocorrosion^60,61^. In this study, the z-scores may look similar in EC-1 (21 genes) and EC-3 (19 genes) at 0.02 each. But most of the crucial genes in both these conditions showed almost opposite gene expression patterns that are related to chemotaxis and two-component systems. Among them, the chemotaxis protein, *cheW* proteins such as Dde_0575 (2.2-folds) and Dde_2040 (0.9-folds) showed up-regulation in SLG-Cu (EC-3), respectively. Whereas in control EC-1, the same proteins Dde_0575 (− 1.2-folds) and Dde_2040 (− 2-folds) were down-regulated, respectively. In addition, the *cheC* domain protein (Dde_1196) was up-regulated in EC-1 (2.6-folds) which showed down-regulation in EC-3 (− 1.2-folds). These *cheW* proteins are involved in the commutation and transmission of sensory signals from chemoreceptors to flagellar motors that aid in bacterial locomotion^62^. In addition, the methyl-accepting chemotaxis sensory transducer proteins were regulated differentially in EC-1 and EC-3. These proteins Dde_1077 (1.5-folds), Dde_0369 (3.4-folds), and Dde_3508 (4.6-folds) were up-regulated in control EC-1. Whereas the same chemotaxis proteins Dde_1077 (− 1-folds), Dde_0369 (− 1.4-folds) and Dde_3508 (− 1.9-folds) were down-regulated in EC-3. These methyl-accepting chemotaxis proteins are receptors present in the cytoplasmic membrane and are involved in bacterial adaptation to diverse stress, cell survival biodegradation, and signal transduction^63^. Similarly, the histidine kinase protein, Dde_2411 (2.5-folds) was also found to be up-regulated in control EC-1, whereas the same protein was down-regulated in EC-3 (− 1.8-folds). Histidine kinase proteins are involved in bacterial sensing and responses to environmental stimuli through a conserved assembly of transmembrane chemoreceptors along with the *cheW* protein^62^.

#### Stress response genes

Bacteria adapt to any potential danger of oxygen metabolism through reactive-oxygen species (ROS) generation that can damage cellular components such as proteins, DNA/RNA, and lipids^64^. To withstand the stress imposed the bacteria defends using superoxide dismutase and peroxides to remove hydrogen peroxide to continuously neutralize the endogenously produced ROS^65^. In this study, the superoxide reductase (Dde_3193) and superoxide dismutase (Dde_0882) were down-regulated in control (EC-1) with -3.3-folds and -1.2-folds, respectively. Whereas the superoxide reductase protein was up-regulated in SLG-Cu, EC-3 (2-folds) revealing that the graphene-coated Cu is triggering the stress-responsive genes involved in ROS generation. This also corroborates with another stress-responsive gene called thioredoxin reductase Dde_2066 which was down-regulated in control EC-1 (− 1.8-folds) but was found to be up-regulated in EC-3 (0.6-folds). Furthermore, the alkyl hydroperoxide reductase/ thiol specific antioxidant protein, Dde_0713 was also found to be down-regulated in control, EC-1 (− 1.3-folds) whereas the same gene was up-regulated in EC-3 (1.9-folds). These results were also in contradiction with the previous result on OA-G20 exposed to 15 µM Cu (II) which showed most of these stress-responsive genes were down-regulated^13^.

### Gene ontology analysis of DEGs

**Figure 2:**
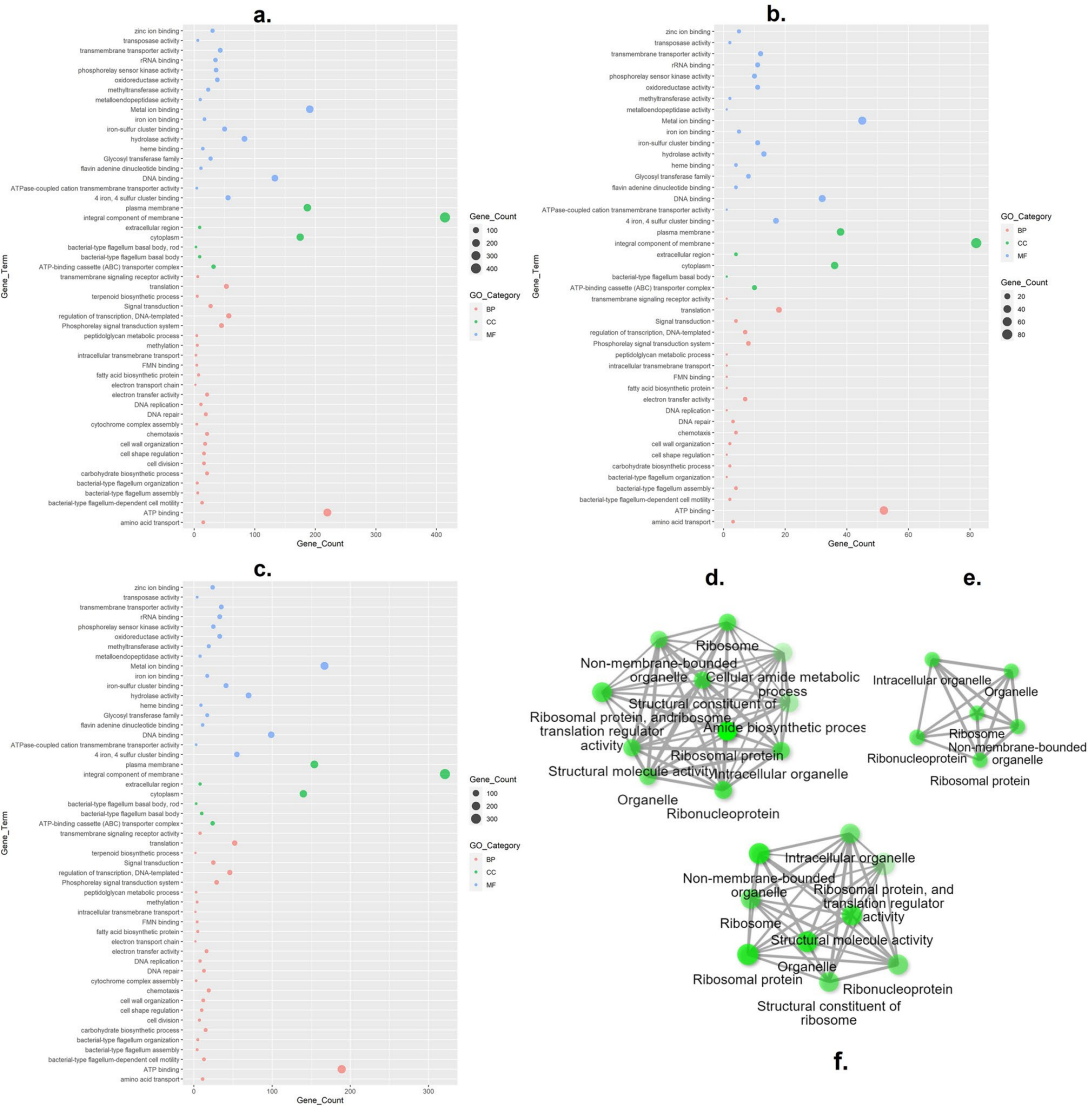
Gene Ontology bubble plot illustrating the enriched GO terms and their corresponding gene counts in OA-G20. (**a**) Control vs. P-Cu (EC-1), (**b**) P-Cu vs. SLG-Cu (EC-2) (**c**) SLG-Cu VS control (EC-3). *BP* biological process; *CC* cellular component; *MF* molecular function. (**d**–**f**) Gene clustering by PPI of in-house generated data of OA-G20 exposed to copper stress (**d** EC-1; **e** EC-2 and **f** EC-3). Each green circle denotes the nodes (genes) that are highly connected by edges (grey arrows).

GO classification revealed the functional correlation between the number of genes enriched from the crucial GO terms. Interestingly the top six enriched GOs in EC-1 (Fig. 2a), EC-2 (Fig. 2b), and EC-3 (Fig. 2c) were an integral component of membrane (GO:0016021), ATP binding (GO:0005524), metal ion binding (GO:0046872), plasma membrane (GO:0005886), cytoplasm (GO:0005737) and DNA binding (GO:0003677). Furthermore, the total gene counts of individual GOs under three experimental conditions were listed in Supplementary Table S2 A-C. The total gene counts for each of the GOs were very high in EC-1 and EC-3 compared to EC-2. The proteins corresponding to these GOs could be related to the increased stability of SRBs under copper stress or translational regulatory mechanisms.

### Gene clustering analysis of DEGs

The PPI network of in-house generated data showed a cluster of genes with highly interconnected nodes formed in samples EC-1, EC-2, and EC-3 (Fig. 2d–f, respectively) showing the three unique clusters. Interestingly, gene clustering analysis revealed that significant interactions occurred between the cellular components such as membrane-bounded organelle genes and ribosome-related pathways in EC-1, EC-2, and EC-3. Also, these strong interactions among them indicate the co-expression systems involved in the different experimental conditions. Generally, the membrane-bound enzymes are the building blocks of the sulfate-reducing bacteria under stress and regulate the organization and structure to retain the membrane fluidity^66^. This result corroborates with the previous study on biocide stress on sulfate-reducing bacteria *D. vulgaris* showing the highest number of 17 up-regulated genes in the translation, ribosomal structure and biogenesis GO^67^. Also, these results are in good agreement with OA-G20 exposed to soluble 10 µM copper with the translation pathways being the strongly interacting gene clusters^13^. Translational regulation of environmental adaptations such as stress response is highly complex that depends on the crucial regulatory steps including start-site selection during transcription, transcriptional coupling, and translation^41^. Besides strong interactions were observed in the cellular amide metabolic process and amide biosynthetic pathways in EC-1. Similar results were observed when *D. vulgaris* was exposed to acidic stress resulting in the up-regulation of amide biosynthetic genes^68^.

### Transcriptomic analysis of the training datasets

#### DGE analysis in training datasets

*Training dataset 1:* Transcriptomic analysis of *M. hydrothermalis* exposed to hydrostatic stress. The DGE analysis revealed that 1404, 874, and 599 genes were differentially expressed in EC-4, EC-5, and EC-6 conditions, respectively (Fig. 1b). In addition, 53%, 42%, and 47% of genes were up-regulated in EC-4, EC-5, and EC-6 conditions, respectively. Whereas 47%, 58%, and 53% were down-regulated in EC-4, EC-5, and EC-6 conditions, respectively. *Training dataset 2:* Transcriptomic analysis of *P. piezophilus* exposed to hydrostatic stress. The DGE analysis revealed that 119, 479, and 533 genes were differentially expressed in EC-7, EC-8, and EC-9 conditions, respectively, (Fig. 1c). In addition, 61%, 57%, and 44% of genes were up-regulated in EC-7, EC-8, and EC-9 conditions, respectively. Whereas 39%, 43%, and 56% were down-regulated in EC-7, EC-8, and EC-9 conditions, respectively. *Training dataset 3:* Transcriptomic analysis of *D. vulgaris* exposed to CuO. The DGE analysis revealed that 140, 92, and 154 genes were differentially expressed in EC-10, EC-11, and EC-12 conditions, respectively (Fig. 1d). In addition, 62%, 35%, and 45% of genes were up-regulated in EC-10, EC-11, and EC-12, respectively. Whereas 38%, 65%, and 55% were down-regulated in EC-10, EC-11, and EC-12, respectively. *Training dataset 4: D. vulgaris* exposed to free nitrous acid (FNA) stress. The DGE analysis revealed that 2331 genes were differentially expressed in EC-16 among which 51% and 49% were up and down-regulated, respectively.

#### Training dataset 1: *M. hydrothermalis* exposed to hydrostatic stress

**Figure 3:**
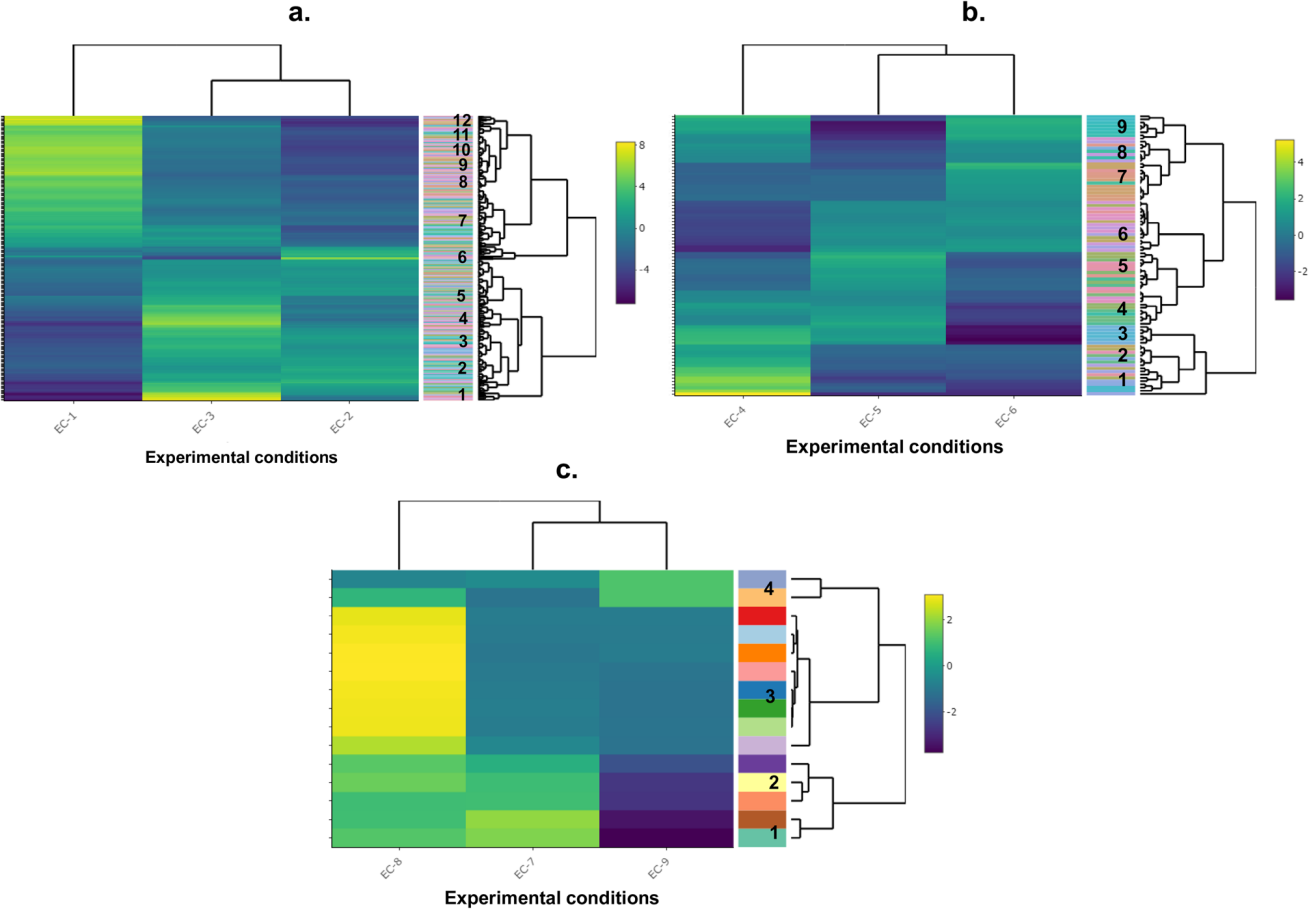
Heat map analysis showing the DEGs of (**a**) OA-G20 exposed to copper stress (EC-1 to EC-3) (**b**) *M. hydrothermalis* exposed to hydrostatic stress (EC-4 to EC-6) (**c**) *P. piezophilus* exposed to hydrostatic stress (EC-7 to EC-9). Genes were selected from the original annotated file with the P-value < 0.05. Gene clusters were also identified from the dendrograms representing the coexpressed genes. The color in the heatmap represents the log2FC and the extra column next to the dendrograms represents the unique genes.

The GO bubble plot revealed the enriched GOs, and their total gene counts in EC-4, EC-5, and EC-6 were tabulated in Supplementary Figs. S1, S2, and S3, respectively. In addition, the top six enriched GOs and their total gene counts in EC-4, EC-5, and EC-6 were tabulated in Supplementary Table S2D–F, respectively. Interestingly, the top six enriched GOs are integral components of membrane, cytoplasm, ATP binding, metal ion binding, plasma membrane, phosphorelay signal transduction system, and translation in EC-4. Whereas the top six enriched GOs in EC-5 are integral components of membrane, metal ion binding, plasma membrane, cytoplasm, ATP binding, and phosphorelay signal transduction system. Besides, the top six enriched GOs in EC-6 are integral components of membrane, cytoplasm, plasma membrane, metal ion binding, ATP binding, and DNA binding. Similarly, the gene clustering analysis revealed three unique clusters with strong interaction among aminoacid-related pathways (tryptophan, phenylalanine, tyrosine, and histidine), e-e2 ATPase, and ribonucleoproteins. These results clearly show that the integral component of membrane, metal/ ATP binding pathway proteins, and signal transduction machinery are involved in co-regulation when *M. hydrothermalis* exposed to hydrostatic stress. The heat map analysis revealed the DGE pattern of selected 88 genes from EC-4, EC-5, and EC-6 (Fig. 3b.). It was observed that 9 gene clusters were formed among the corresponding experimental conditions.

#### Training dataset 2: *P. piezophilus* exposed to hydrostatic stress

The GO bubble plot revealed the enriched GOs, and their total gene counts in EC-7, EC-8, and EC-9 were tabulated in Supplementary Figs. S4, S5, and S6, respectively. In addition, the top six enriched GOs and their total gene count in EC-7, EC-8, and EC-9 were tabulated in Supplementary Tables S2G–I, respectively. In EC-7, the top six enriched GOs are the integral component of membrane, ATP binding, plasma membrane, metal ion binding, DNA binding, and extracellular region. The top six enriched GOs in EC-8 are the integral component of membrane, plasma membrane, cytoplasm, translation, ATP binding, metal ion binding, and rRNA binding. The top six enriched GOs in EC-9 are integral components of membrane, cytoplasm, plasma membrane, metal ion binding, ATP binding, and translation. Besides the gene clustering analysis showed that a strong network of three unique gene clusters was formed in EC-7. These include a gene cluster among membrane (transmembrane, methyltransferase, and thioesterase), transport (zinc, hyld family secretion protein, and rnd efflux pump), organelle (flagellum, cilium, and secretory pathways). Whereas in EC-8, three distinctive gene clusters were formed and include ribosomal (ribonucleoprotein, rRNA binding, protein biosynthesis, and elongation factor), secretion system (glycosyl transferase family-2 and type II secretion pathways), and transport system (ABC transporter transmembrane region and alpha/beta hydrolase proteins). Similarly in EC-9, three unique clusters were interconnected that include ribosomal (ribonucleoprotein, rRNA binding, protein biosynthesis, and elongation factor), chemotaxis (HAMP domain, transducer protein, and methyl-accepting chemotaxis protein). These results indicate that the *P. piezophilus* under hydrostatic stress results in the co-expression of transport domains, membrane proteins, fatty acid biosynthesis secretory, and chemotaxis pathways. The heat map analysis revealed the DGE of selected 15 genes from EC-7, EC-8, and EC-9 (Fig. 3c.). It was observed that 4 gene clusters were formed among the corresponding experimental conditions.

#### Training dataset 3: *D. vulgaris* exposed to CuO

The GO bubble plot revealed the enriched GOs, and their total gene counts in EC-10, EC-11, and EC-12 were tabulated in Supplementary Figs. S7, S8, and S9, respectively. In addition, the top six enriched GOs and their total gene count in EC-10, EC-11, and EC-12 were tabulated in Supplementary Table S2J–L, respectively. The top six GOs enriched in EC-10 are integral components of membrane, metal ion binding, plasma membrane, signal transduction, cytoplasm, and ATP binding. Whereas the top six GOs enriched in EC-11 are integral components of membrane, plasma membrane, metal ion binding, cytoplasm, 4 iron, 4 sulfur cluster binding, DNA binding, extracellular region, and electron transfer activity. Besides EC-12, the top six enriched GOs are integral components of membrane, plasma membrane, metal ion binding, cytoplasm, 4 iron, 4 sulfur cluster binding, and ATP binding. In addition, the gene clustering analysis of EC-10 revealed two unique clusters with strong interconnecting nodes including flagella (flagellin, flagellar assembly, flagellar rotation, flagellar biogenesis, and cilium) and chemotaxis (methyl-accepting chemotaxis protein and transducer). Whereas in EC-11, two small gene clusters were formed and that includes cytochrome and DNA binding domain proteins. Interestingly, in EC-12 a unique gene cluster was formed with 17 different genes that predominantly deal with ATP biosynthesis, hydrogen ion transport, proton transport, ion channel activity, and purine nucleoside triphosphate biosynthesis. This unique cluster indicates that the genetic regulation under high CuO concentration resulted in huge genetic components being co-regulated compared to other conditions. Overall, the hydrostatic stress to *D. vulgaris* played a major co-regulation of transport proteins (ion, proton), transcriptional regulators ATP biosynthesis, and chemotaxis.

#### Training dataset 4: *D. vulgaris* exposed to FNA stress

The GO bubble plot revealed the enriched GOs, and their total gene counts in EC-13 were tabulated in Supplementary Fig. S10. In addition, the top six enriched GOs and their total gene count in EC-13 were tabulated in Supplementary Table S2M. The top six GOs enriched in EC-13 are integral components of membrane, ATP binding, cytoplasm, metal-ion binding, plasma membrane, and DNA binding. The gene clustering analysis revealed a unique strong interconnected cluster with 16 different genes. These genes are mainly involved in ion channel activity, proton transport, purine biosynthesis, and ATP biosynthesis. This data indicates that *D. vulgaris* under FNA stress resulted in the GO enrichment and co-regulation of stress response genes involved in ATP/proton regulation, flagellar pathways, transcription, and translation regulatory pathways.

### Crucial pathways of SRBs triggered under stress

Nowadays, the traditional anti-corrosive coatings to prevent biofilm formation are primarily inefficient against biofilm microstructures as they are profoundly evolving to become more metal or antibiotic-resistant which further complicates the issue^69^. Previously, the gene regulatory pathways related to energy metabolism, formate cycling, -osmo protection, reactive oxygen species (ROS) protection, and iron homeostasis were observed to be the crucial components for SRBs under stress^26^. But with the advent of integrated and coordinated efforts including OMIC approaches such as transcriptomics, proteomics, and single-cell genomics, the in-depth genetic anomalous behaviors of SRBs could be unraveled^26^. Recent studies have demonstrated the transcriptome-based differential gene transcript profiles of SRBs exposed to various environmental stress conditions^9,33–36^. Based on this study, the crucial genetic pathways observed were chemotaxis, flagellum, signal transduction, ATP biosynthesis, glycosyl transferase, transcriptional regulation, membrane transport, ribonucleoproteins, and secretion systems.

Chemotaxis is a biological process by which the bacteria sense and responds to any changes in the environmental conditions by their metabolic regulations by moving away from or toward the changing environmental stimuli^70^. This switching mechanism is mainly activated/ repressed by the sensory signals that regulate the release of small-phosphorylated response regulators which bind to the rotary flagellar motor. Depending upon the concentration of the response regulators, the gene expression of the flagellar-associated proteins is up/down-regulated and thus chemotaxis and flagellar assembly pathways are related^71^. In addition, the reduced cellular motility of SRB and chemotaxis activity is reported to be a crucial mechanism in SRB heavy-metal stress response^9^. Signal transduction is another critical pathway involved in combating the stress response in SRBs that harbor genes that are controlled by their interactions among the transcriptional regulators including catalytic core RNA polymerase and sigma factors. These sigma factors are dissociable prokaryotic RNA polymerase subunits that regulates crucial pathways including stress tolerance, iron uptake, outer-membrane porins, alginate biosynthesis, and virulent factors expression^72^. In addition, the metal resistance in bacteria is associated with the cellular signaling pathways such as two-component signaling (TCS) systems that allow the microbes to sense, react and fine-tune to signal changes according to the environmental stimuli, intracellular conditions, quorum sensing signals, antibiotics, osmotic stress, and cellular redox environments^73^. These signal transduction pathways are a part of intracellular signal processing that mediates the environmental stimuli and the specific adaptive responses which include flagellar proteins, methyl-accepting chemotaxis, and cyclic-diguanosine monophosphate (c-di-GMP)-related proteins^74^. Interestingly, the σ^54^-dependent regulons are associated with the type III secretion systems, electron transfer, pyruvate transport metabolism, and alanine dehydrogenase of *D. vulgaris*^75^.

### Overlapping genes and comparative genomics

**Figure 4:**
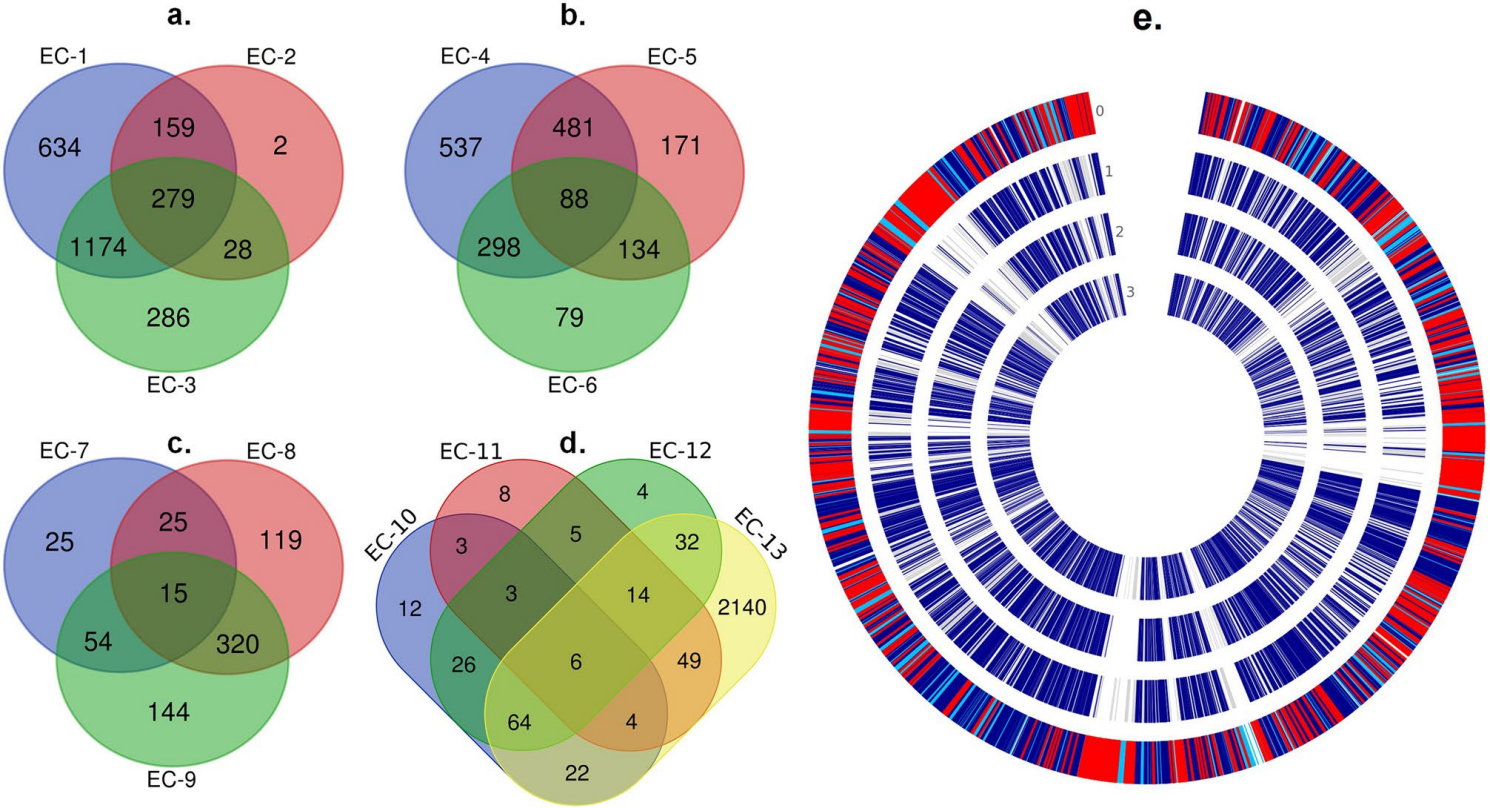
(**a**–**d**) Venn diagram representing the distribution of intersecting DEGs in the dataset collections (EC-1 to EC-13). **a**. OA-G20 exposed to copper stress **b**. *M. hydrothermalis* exposed to hydrostatic stress **c**. *P. piezophilus* exposed to hydrostatic stress and **d**. *D. vulgaris* exposed to CuO and FNA stresses. (**e**) Comparative genome analysis (core, non-core, and base-singletons) of four SRBs used in this study shows the orthologous genes among them. Genome 0: OA-G20, Genome 1: *D. vulgaris* Hildenborough, Genome 2: *P. piezophilus* C1tlv30, Genome 3: *M. hydrothermalis* AM13; dark blue—core pangenome; grey—non-core pangenome; red—base singletons (OA-G20); blue—non-core (OA-G20).

Gene co-expression was analyzed using the Venn diagram (Fig. 4a-d) that revealed the numbers of DEGs across all four SRBs under 13 experimental comparisons and the overlapping genes by intersection and union that is involved in the corresponding stress conditions of the SRBs evaluated in this study. The results showed that 279, 88, 15, and 6 genes were observed to be the intersecting genes among all the experimental conditions of OA-G20 (Fig. 4a), *M. hydrothermalis* (Fig. 4b), *P. piezophilus* (Fig. 4c)*,* and *D. vulgaris* (Fig. 4d), respectively. These genes are attributed to the uniquely expressed genes among the SRBs under stress conditions. The circle plot (Fig. 4e) analysis showed the comparative genome of four SRBs analyzed in this study. the core, non-core, and base singleton genes among all the four SRBs evaluated in this study. This showed that 12,821 genes with translation, 10,178 are in homolog families and 2643 genes were in singleton families. In addition, a total of 5773 gene cluster families have been identified among all four SRBs. Also, among them, 3130 homolog families and 2643 singleton families were identified (Supplementary Table S3.). Furthermore, the numbers of homologous genes among the SRBs were also listed in Supplementary Table S4. Further comparative genomics studies including pangenome analysis of these SRBs will reveal more genomic insights beyond the orthologues and paralogues genesets which will aid the biomarker discovery for stress-responsive genes in SRBs.

### Key findings and future directions

A crucial contribution of this study is the integrated transcriptomic approach that can be used to analyze the genotypical behavior of different SRBs exposed to different types of stressors. The current study focused on four SRBs exposed to four different stressors (details in Table 1) including the copper stress induced by the presence of graphene on copper (Cu) substrates. The OA-G20 cells exposed to SLG-Cu were found to uniquely up-regulate dinitrogenase iron-molybdenum cofactor biosynthesis protein (Dde_3198 and Dde_3197) compared to control. The protein sequence homology analysis of Dde_3198 and Dde_3197 revealed that the sequence homology aligns with other dinitrogenase iron-molybdenum cofactor biosynthesis proteins of phylogenetically related *Desulfovibrio* sp. On the other hand, the translation machinery in OA-G20 was remarkably up-regulated in P-Cu and SLG-Cu exposure to withstand stress induced by the environmental stimuli. The PPI network of the in-house generated data showed a cluster of genes with highly interconnected nodes showing the three unique clusters in EC-1, EC-2, and EC-3. The gene clustering analysis revealed significant interactions among the cellular components (e.g., membrane-bounded organelle genes) and ribosome-related pathways. Furthermore, the comparative genomic analysis revealed the core, non-core, and base singleton genes among all the four SRBs evaluated in this study. This analysis showed 12,821 genes with translation, 10,178 genes in homolog families, and 2643 genes in singleton families. This analysis could help in the recognition of unknown regulatory regions in their genomes. This study summarizes the evidence-based transcriptome datasets to develop a meta-analysis platform for large gene expression datasets of SRBs. This ensures that the RNA-seq data analyzed by multiple different labs can be combined to provide more meaningful and comprehensive information on stress responses and environmental adaptation of different SRBs. These data will also integrate the genetic co-virulence factors leading to stress-based metal/ antibiotic resistance. Nonantibiotic compounds including metals and biocides are the major drivers for the stress response stimuli among the sensitive bacteria resulting in antibiotic resistance development through co-selection. This study thus advances the current state of knowledge towards the meta-analysis approach to understand the deeper inter-study variations, gene clustering features through comparative genomics. This way, a new research direction can be set to develop the integrated machine-learning-enabled transcriptomic atlas for SRB stress response.

## Materials and methods

### Cultivation of OA-G20 under copper stress and RNA extraction

The OA-G20 strain was a generous gift from Dr. Mathew Fields, Professor, and Director at Center for Biofilm Engineering, Montana State University, Bozeman, MT, USA. This strain was originally purchased from Leibniz Institute DSMZ—German Collection of Microorganisms and Cell Cultures GmbH, Germany. This strain was also identified and confirmed using 16S rRNA gene sequencing analysis. Initially, the genomic DNA was extracted from the exponential phase cultures, and the 16S rRNA gene sequences were PCR amplified using 8F and 1492R universal primers. The amplified PCR fragments were sequenced and confirmed. The pure cultures of OA-G20 seed cultures were sub cultured from 1 mL of frozen stock (50% v/v glycerol stock) to 10 mL of freshly prepared modified 1actate-C medium^76^. This media comprises the following constituents (g/L): sodium lactate—6.8, sodium citrate—0.3; sodium sulfate—4.5, calcium chloride dehydrated—0.06, ammonium chloride—1, magnesium sulfate—2, potassium phosphate monobasic—0.5; yeast extract—1 with pH adjusted to 7.2. An exponential phase of these cultures was used for further experimental study. The bacterial cell densities were evaluated by measuring the optical density at 600 nm using BioTek Epoch 2 Microplate Spectrophotometer (Agilent). The optical densities of the initial inoculum used were 0.05. The cell concentrations were observed to reach a maximum optical density of 0.15. A 10% initial inoculum was used with 100 mL of total culture final volume. Three different experimental conditions were used, including: 1. no copper (EC-1), 2. P-Cu (EC-2), and 3. SLG-Cu (EC-3). A PTC1 PortHoles Electrochemical Sample Mask (Gamry Instruments Part No. 990/00254) was used as the masking tape to expose only the specified surface area of coupons. Also, the OA-G20 cultures were exposed to only one side of the coupon with a consistent exposure surface area of 1 cm^2^. The other side of the coupon was masked to prevent OA-G20 exposure. The pristine copper foil (P-Cu) was 0.025 mm (0.001in) thick was purchased from Alfa Aesar Puratronic (7440-50-8). Whereas the Single Layer Graphene on Copper Foil (SLG-Cu) was purchased from Graphene Super Market (SKU: CVD-CU-4X4). These graphene films were grown using Chemical Vapor Deposition (CVD) process on copper foil and these coatings are continuous across copper surface steps and grain boundaries. Before the serum bottle tests, these materials were pretreated by cleaning them with deionized water followed by ethanol and air drying. The coupons of P-Cu and SLG-Cu materials were circular in shape which were attached on to a glass slide (2.5 cm × 1 cm) using an electrochemical PTC1 PortHoles Electrochemical Sample Mask tape. The total exposure area of the coupons of 1 cm^2^ was maintained in both P-Cu and SLG-Cu. The controls (no treatment) did not have any coupons. The coupons were inserted into 150 mL serum bottle containing 90 mL of lactate C growth medium (pH 7.2). The bottles were tightly crimp-sealed with butyl rubber stoppers and aluminum caps. The sealed serum bottles were then purged with nitrogen gas for 20 min to create an anaerobic condition. The cultures were incubated at 30 °C under static conditions for 7 days. After incubation, the planktonic cells were harvested by centrifuging at 12,000×*g* for 10 min at 4 °C. The cell pellets were washed twice using phosphate buffer saline (pH 7.2) to remove the media salts. The total RNA was extracted using TRIzol Max Bacterial RNA Isolation Kit (Ambion, Life Technologies) by following the manufacturer’s protocol. Additionally, the RNA was purified using the RNA clean and concentrator kit following the manufacturer’s instructions (Zymo Research, Irvine, CA, USA). The quality, quantity, and integrity of the RNA samples were evaluated using Qubit 3.0 fluorometer (Thermo Fisher Scientific, Waltham, MA, USA) and nanodrop (Nanodrop™ 1000 spectrophotometer, Thermo Scientific). RNA samples were shipped to the Oklahoma Medical Research Foundation (Oklahoma City, OK, USA) for RNA-seq analysis. RNA-seq datasets were analyzed thoroughly and advanced multi-omics computational techniques were utilized. The steps involved in the library preparation and RNA-sequencing are as follows: rRNA depletion, cDNA library preparation, and RNA-sequencing. Initially, rRNA depletion was carried out using RiboCop rRNA Depletion Kit (Lexogen, Greenland, NH, USA) following the manufacturer’s protocol. By this, large amounts of undesired transcripts were removed to access the transcripts of interest. This includes the coding mRNA transcripts that afford the organism’s complete transcriptome. Then cDNA library was prepared using Swift Rapid RNA Library Kit (Swift Biosciences, Ann Arbor, MI, USA) following the manufacturer’s protocol. Here the ribosomal RNA-depleted RNA was then fragmented followed by random priming and reverse transcription to generate the first-strand cDNA. i5 and i7 adapters were then incorporated followed by indexing PCR performed that resulted in full-length adapters. Furthermore, Kapa qPCR and Agilent Tapestation 4150 (Agilent Technologies, Santa Clara, CA, USA) were used to prepare for the final quality control. Finally, the Illumina-NovaSeq 6000 platform using an S4 flow cell with a 150-bp paired-end module was used to sequence the libraries constructed. Nearly 20 million reads (10 million in each direction) were generated per replicating samples. All the RNA-sequencing samples were performed in triplicates.

### RNA-seq dataset collection and analysis

Publicly available gene expression datasets of SRBs exposed to stress were examined in the databases including Gene Expression Omnibus (GEO) and Sequence Read Archive (SRA) that are maintained by National Center for Biotechnology Information (NCBI)^77,78^. BioProjects (Table.1) were selected based on several criteria including the number of samples tested, type of stressors, sequencing layout (paired or single end), and the sequencing platform. This study precisely focused on generating the datasets that can be used to reveal transcriptomes of SRB cells exposed to stress conditions. In general, the number of RNA-seq datasets for SRBs exposed to stress conditions is scanty. The goal of this study was to identify candidate gene lists of SRBs exposed to different stressors. Relevant keywords were framed to return the needed datasets for SRBs exposed to various stress conditions. The RNA-seq datasets of *Desulfovibrio* sp. available in NCBI GEO and ArrayExpress until October 2021 were curated and analyzed in this study. A specific focus was on generating gene expression datasets obtained using the Illumina sequencing platform. The following eight criteria were used to mine relevant BioProjects identified from PUBMED, Array Express, and NCBI GEO. This criterion included: 1. Must be a sulfate-reducing bacteria; 2. Must have a BioProject ID, SRA, and NCBI GEO IDs; 3. Must be grown under stress conditions (e.g., metals, high pressure, acids, or nanoparticles); 4. Must have comparable conditions with control; 5. Must be performed using single or paired-end RNA-sequencing layout; 6. Must have complete raw data and the processed RNA-seq data; 7. Must pass the FAST-QC quality control in GALAXY; and 8. Must possess a minimum of one sample tested with a control condition (at least 1 treatment compared with 1 control). In addition, the major goal of this work is to capture the gene expression pattern and potential gene candidates involved in stress responses of SRBs and thus datasets with and without replicates were considered. On another note, hydrostatic stress acts as a major driving factor for the SRBs to cause MIC in the mining and civil infrastructures^33,34,79–81^. In addition, due to the antimicrobial properties of the free nitrous acids, they were reported to alleviate the hydrogen sulfide production by SRBs^36^. Thus, these two stressors of high pressure and FNA were remarkably suitable driving forces for the environmental stress response and adaptation.

### RNA-seq data analysis

The bioinformatics pipelines and data analysis were performed using a web-based scientific assessment platform called Galaxy^82^. The raw RNA-seq reads were subjected to QC analysis using the FASTQC tool in GALAXY which generates the average Q-scores through all the sequence files^83^. The quality-checked reads were then trimmed for adapters and linkers using the Trimmomatic tool (Galaxy version 2.11)^84^. Sliding window filtration was used to further trim the raw reads to shorten the reads with a Q-score of 20 or below at a 4-base average and reads greater than 20 bp were retained^13^. Furthermore, the QC reads were mapped against the corresponding reference genomes (OA-G20—NCBI Reference sequence: NC_007519.1) using the HISAT2 (Galaxy Version 2.2.1 + galaxy1) alignment tool^85^. The other SRBs evaluated, and their accession numbers were *Maridesulfovibrio hydrothermalis* (PRJNA240876), *Pseudodesulfovibrio piezophilus* (PRJNA251861), *Desulfovibrio vulgaris* (PRJNA395924) and *Desulfovibrio vulgaris* (PRJNA314088). The respective reference genome annotation file (GFF3, Ensembl) and the feature counts (Galaxy Version 2.0.1 + galaxy2—read summarization program) tools were used to count each gene in the genome and the number of genes was mapped^86^. DESeq2 package^87^ was used to analyze the DGE in fold change across all the experimental conditions.

### Gene ontology analysis and gene clustering

The data analysis after ratio normalization yielded the gene transcripts with significant DGE (p–value < 0.05 and |log2FC|> 0) were used in further steps. These DEGs were functionally annotated with their corresponding proteins using the UniProt database^88^. Furthermore, these genes were assorted based on three unique gene ontology terms including biological process, molecular function, and cellular component. The statistical programming language R (version 4.1.2) was used for computational analysis, interactive GO plots, and heat maps. The gene sets were envisaged as a hierarchical clustering tree using ShinyGO version 0.76.3^89^. Gene clustering by PPI analysis was performed to analyze the gene panel mapping and clustered genes. To capture the overall impulse towards up or down-regulations of each GO term/gene, a z-score was calculated^90^.

### Genetic co-expression and comparative genomic analysis

The overlapping DEG from the four sets of datasets used in this study was identified using the Venn diagram using Bioinformatics and Evolutionary genomics tool (https://bioinformatics.psb.ugent.be/webtools/Venn/). Here, DEG of statistical significance (P < 0.05) in all 13 experimental conditions evaluated in this study was used for identifying the intersecting genes under the corresponding stress conditions. Comparative genomics analysis was performed among the genomes of all four SRBs evaluated in this study using KBase (https://www.kbase.us/) Predictive Biology tool^91^. Here the FASTA files of the four genomes were imported to the KBase narrative and annotated the corresponding genomes using PROKKA v1.14.5. Furthermore, the comparative genome was calculated using Build Pangenome with OrthoMCL v2.0. Then the comparative genome modeling was visualized using Pangenome Circle Plot v1.2.0.

## Conclusion

This current study puts forwards a consistent computational methodology for evaluating and comparing the transcriptome of the in-house generated data with the publicly available data. This study also identified the crucial transcriptional genes of SRBs that are involved in co-regulation under various stresses. Also, gene clustering analysis revealed the strongly interconnected nodes among the DEGs. The top enriched GOs in most SRBs were found to be integral component of membrane (GO:0016021), ATP binding (GO:0005524), cytoplasm (GO:0005737), plasma membrane (GO:0005886), DNA binding (GO:0003677) and metal ion binding (GO:0046872). This study also identified that proteins related to transcriptional regulation, chemotaxis, flagellum, membrane transport, ATP biosynthesis, secretion systems, and ribonucleoproteins are the essential pathways of SRBs exposed to various stressors. Furthermore, the PPI network revealed the differentially expressed genes and their intricate genetic network. Also, the genomes of four different SRBs evaluated in this study were grown in four different stress conditions and this can also impact specifically on the DEG analysis. This present study contributes to the comprehensive gene candidates that are involved in the highly biodynamic stress-responsive genetic mechanisms of four crucial SRBs. This information is crucial for further studies that involve training the machine learning tools to predict exactly the crucial transcriptional biomarkers that are involved in the stress response mechanisms of SRB. These gene candidates and GO terms that are involved in the co-regulation of SRB’s stress response should be deemed as the signature target to develop advanced materials including protective coatings based on 2D materials for controlling the corrosive effects of SRB. These gene candidates and the GO terms that are involved in the co-regulation of SRB’s stress response and their biological implication of the signature genes involved in heavy metal stress and environmental adaptation.

### Supplementary Information

Supplementary Information.

## Data Availability

The in-house datasets analyzed in this current study are available in the NCBI repository (https://www.ncbi.nlm.nih.gov/) under the BioProject IDs PRJNA1003487. The gene expression datasets were also submitted to the NCBI Gene Expression Omnibus (GEO) under the accession number GSE240798. The BioProject IDs of the public datasets evaluated in this study are mentioned in Table 1. Finally, the processed dataset will be available in our Biofilm Data and Information Discovery System (Biofilm-DIDS)^92^.
