# Supplementary material for: Transcriptome-wide marker gene expression analysis of stress-responsive sulfate-reducing bacteria
Source: Sci Rep. 2023 Sep 27;13:16181. doi: 10.1038/s41598-023-43089-8 (PMC10533852; doi:10.1038/s41598-023-43089-8)
Supplement: Supplementary file 1 — Supplementary Information. [file 41598_2023_43089_MOESM1_ESM.docx]

Supplementary Material

**Transcriptome-wide marker gene expression analysis of stress-responsive sulfate-reducing bacteria**

Kalimuthu Jawaharraj^1,2,3^, Vincent Peta^4^, Saurabh Sudha Dhiman^1,3,5^, Etienne Gnimpieba^2,3,4*^,Venkataramana Gadhamshetty^1,2,3*^

^1^Civil and Environmental Engineering, South Dakota Mines, 501 E. St. Joseph Street, Rapid City, SD, 57701, USA

^2^2D-materials for Biofilm Engineering, Science and Technology (2DBEST) Center, South Dakota Mines, 501 E. St. Joseph Street, Rapid City, SD, 57701, USA

^3^Data-Driven Materials Discovery for Bioengineering Innovation Center, South Dakota Mines, 501 E. St. Joseph Street, Rapid City, SD, 57701, USA

^4^Biomedical Engineering, University of South Dakota, 4800 N Career Ave, Sioux Falls, SD 57107

^5^Chemistry, Biology and Health Sciences, South Dakota Mines, 501 E. St. Joseph Street, Rapid City, SD, 57701, USA

**Corresponding authors:**

*Dr. Etienne Gnimpieba (Etienne.Gnimpieba@usd.edu)

Research Assistant Professor, Department of Biomedical Engineering, University of South Dakota, 414 E. Clark Street, Vermillion, SD 57069, United States

*Dr. Venkataramana Gadhamshetty ([Venkata.gadhamshetty@sdsmt.edu](mailto:Venkata.gadhamshetty@sdsmt.edu))

Professor, Department of Civil and Environmental Engineering, South Dakota Mines

501 E St Joseph St, Rapid City, SD-57701, United States

**
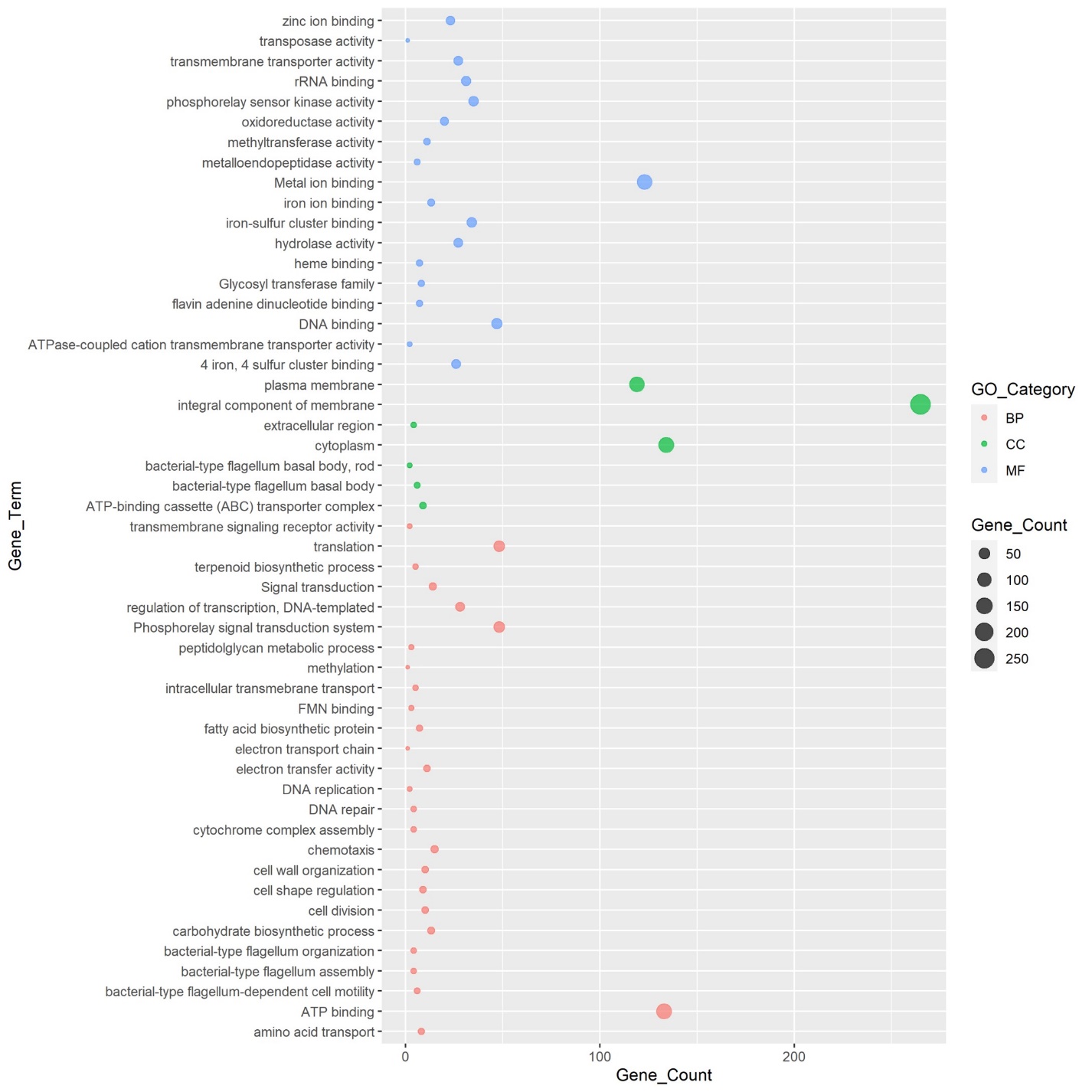
**

**Supplementary figure S1.** Gene Ontology bubble plot illustrating the enriched GO terms and their corresponding gene counts in *M. hydrothermalis* - control vs. 10 MPa (EC-4). BP - Biological process; CC - Cellular component; MF - Molecular function


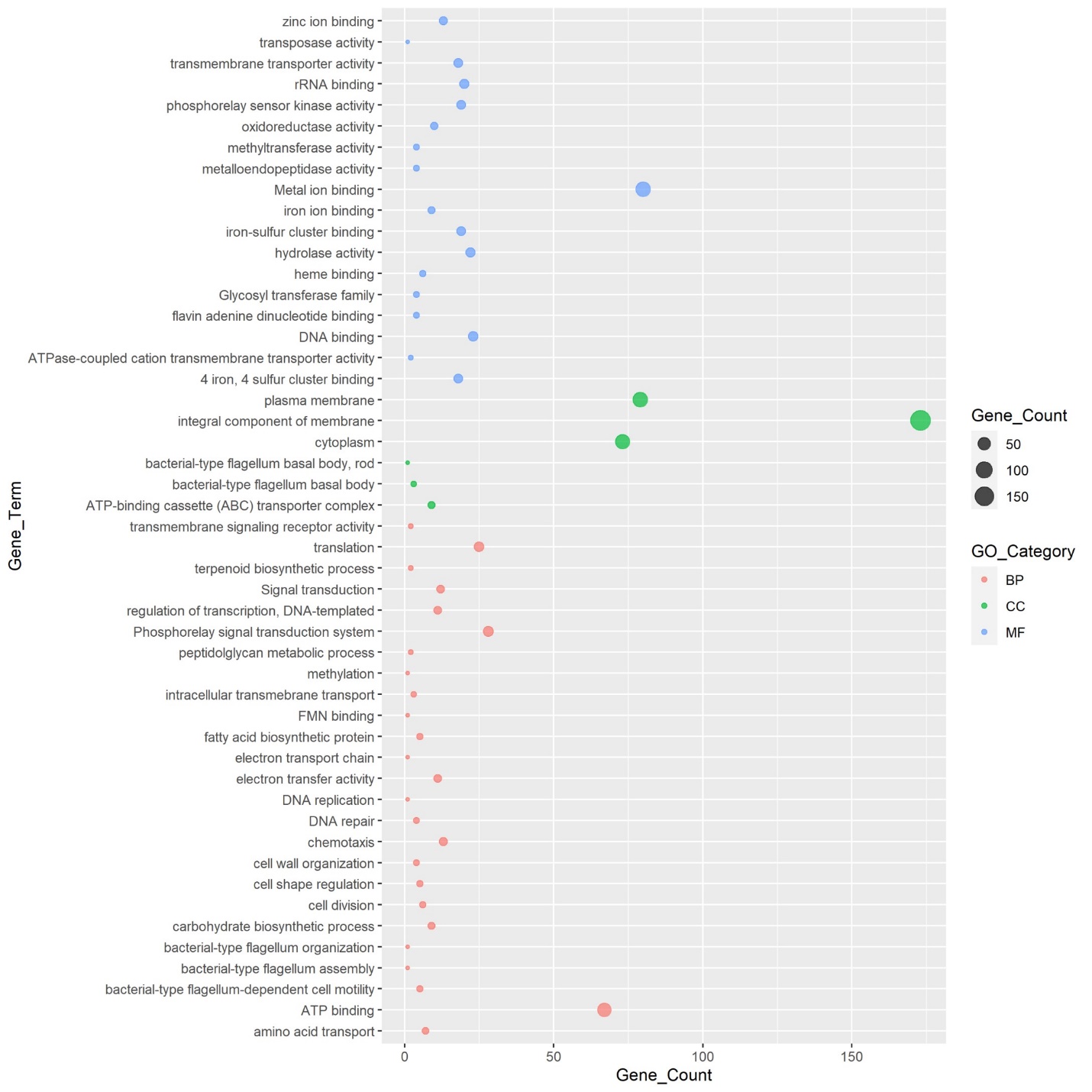


**Supplementary figure S2.** Gene Ontology bubble plot illustrating the enriched GO terms and their corresponding gene counts in *M. hydrothermalis* - 10 MPa vs. 26 MPa (EC-5). BP - Biological process; CC - Cellular component; MF - Molecular function


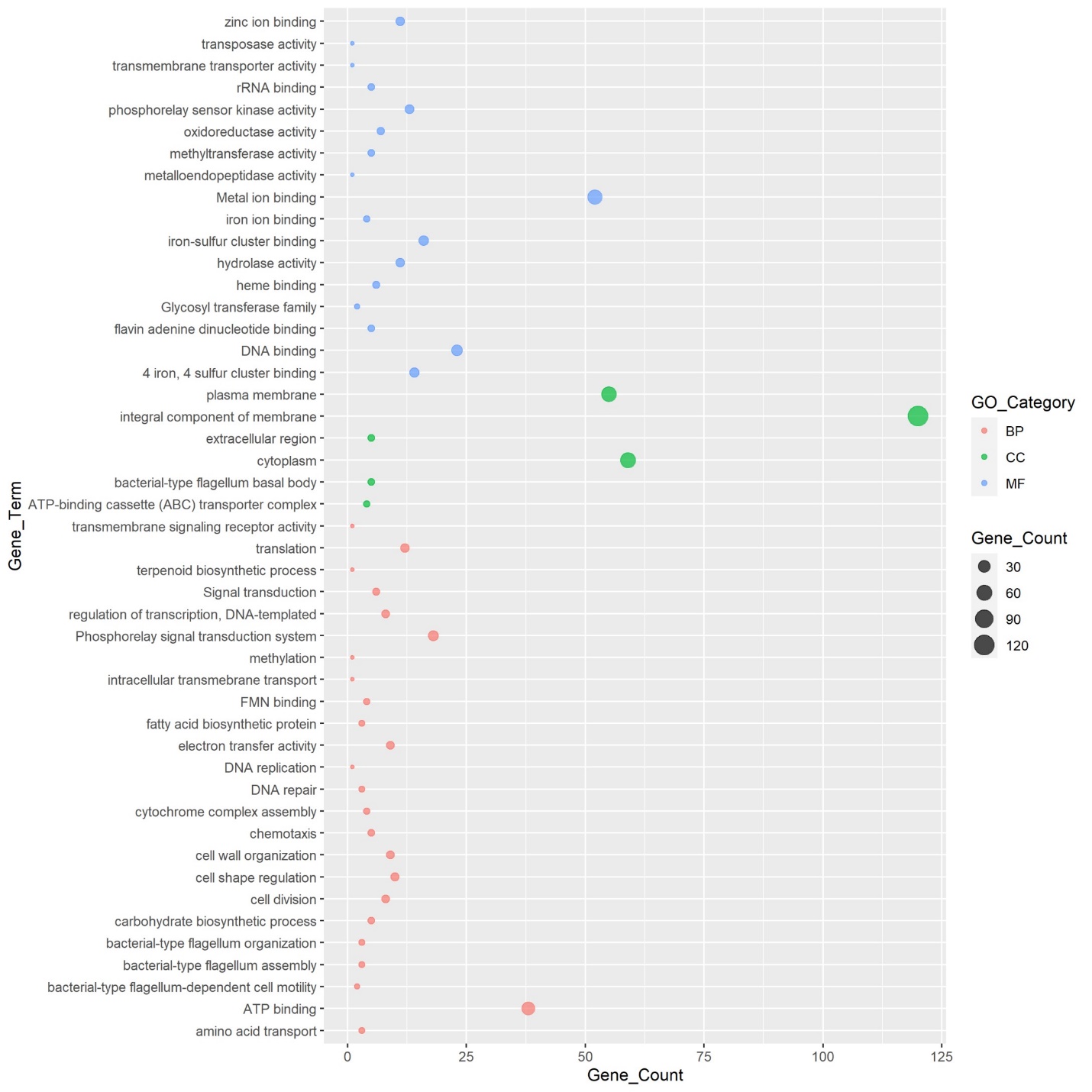


**Supplementary figure S3.** Gene Ontology bubble plot illustrating the enriched GO terms and their corresponding gene counts in *M. hydrothermalis* - 26 MPa vs. control (EC-6). BP - Biological process; CC - Cellular component; MF - Molecular function


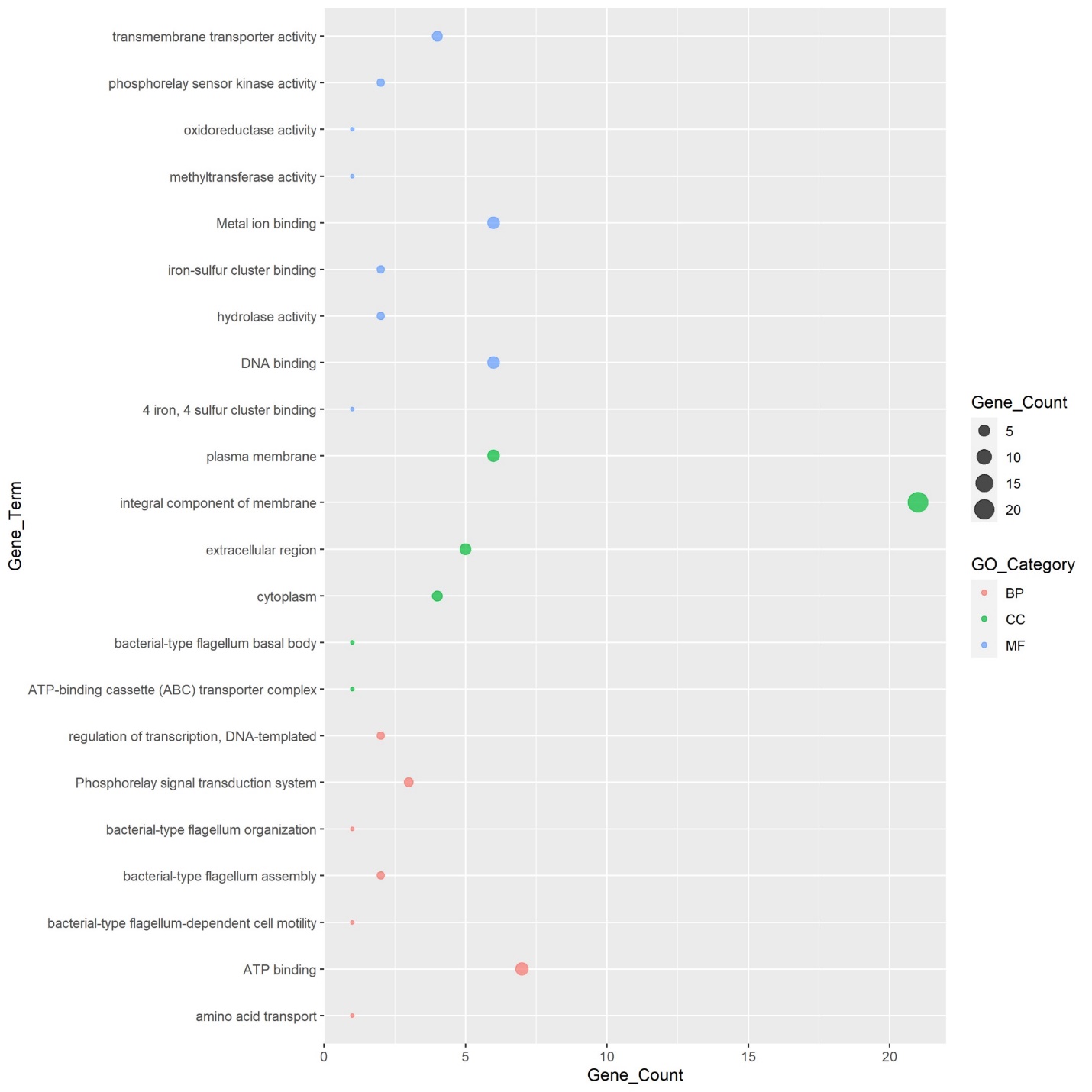


**Supplementary figure S4.** Gene Ontology bubble plot illustrating the enriched GO terms and their corresponding gene counts in *P. piezophilus* – control vs. 10 MPa (EC-7). BP - Biological process; CC - Cellular component; MF - Molecular function


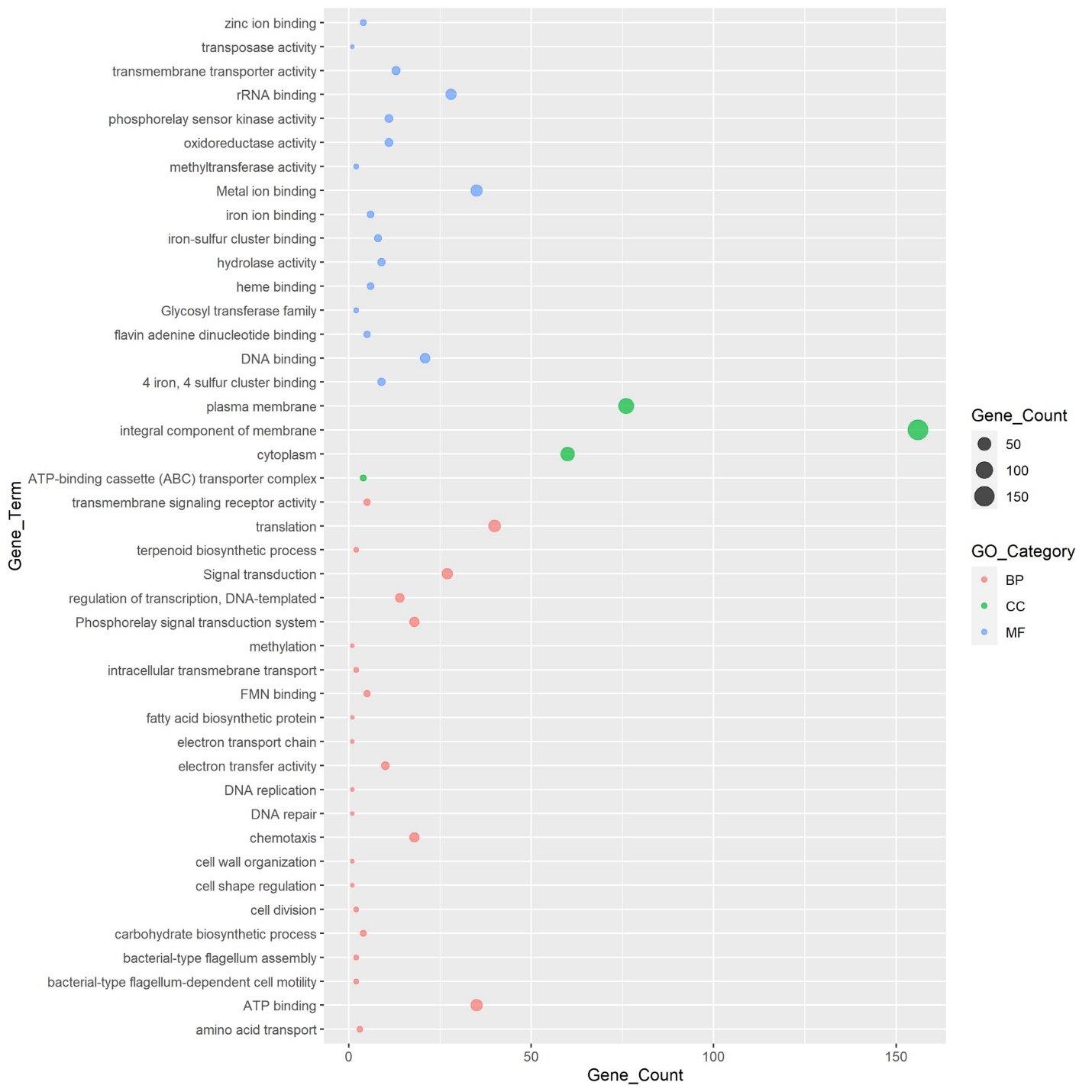


**Supplementary figure S5.** Gene Ontology bubble plot illustrating the enriched GO terms and their corresponding gene counts in *P. piezophilus* – 10 MPa vs. 26 MPa (EC-8). BP - Biological process; CC - Cellular component; MF - Molecular function


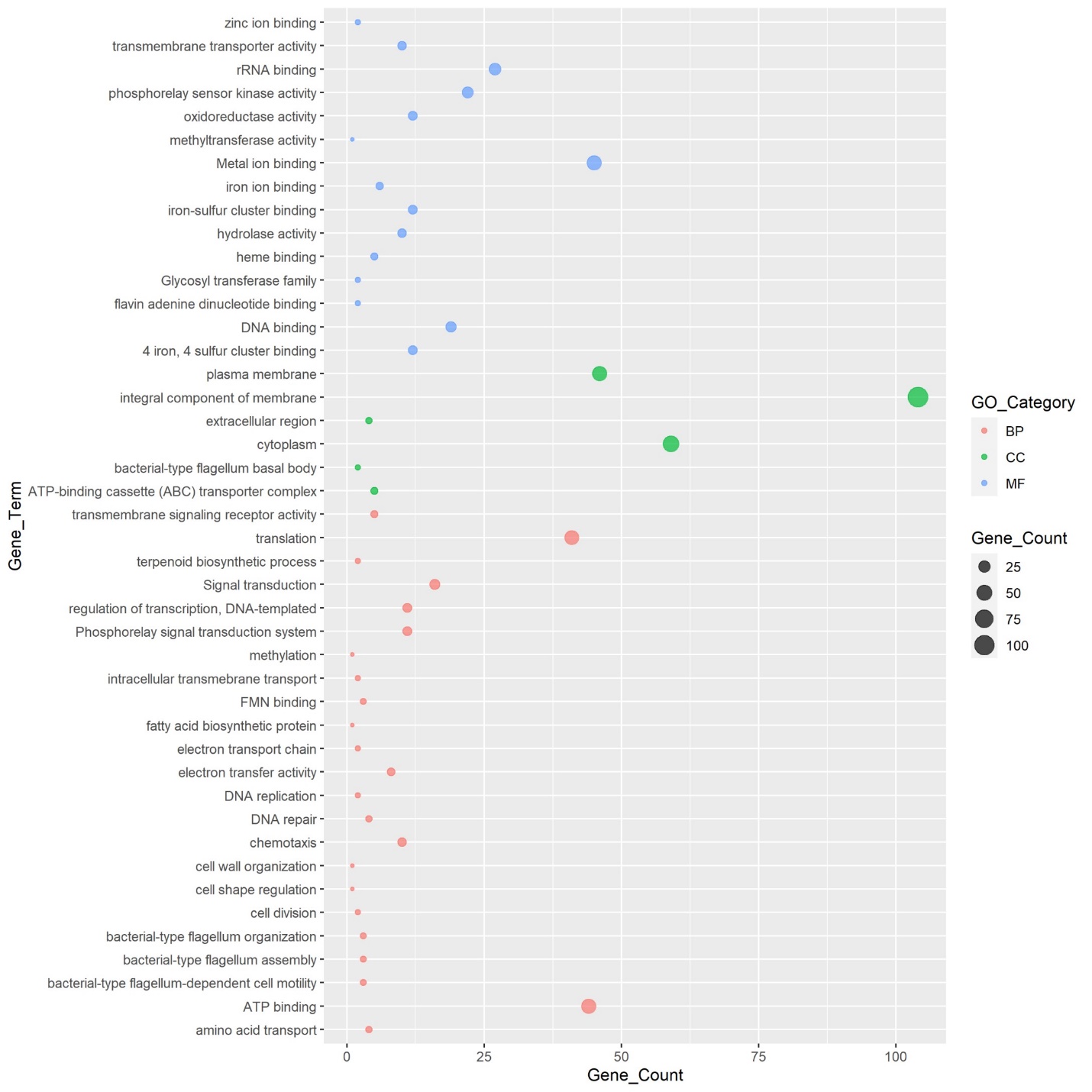


**Supplementary figure S6.** Gene Ontology bubble plot illustrating the enriched GO terms and their corresponding gene counts in *P. piezophilus* – 26 MPa vs. control (EC-9). BP - Biological process; CC - Cellular component; MF - Molecular function


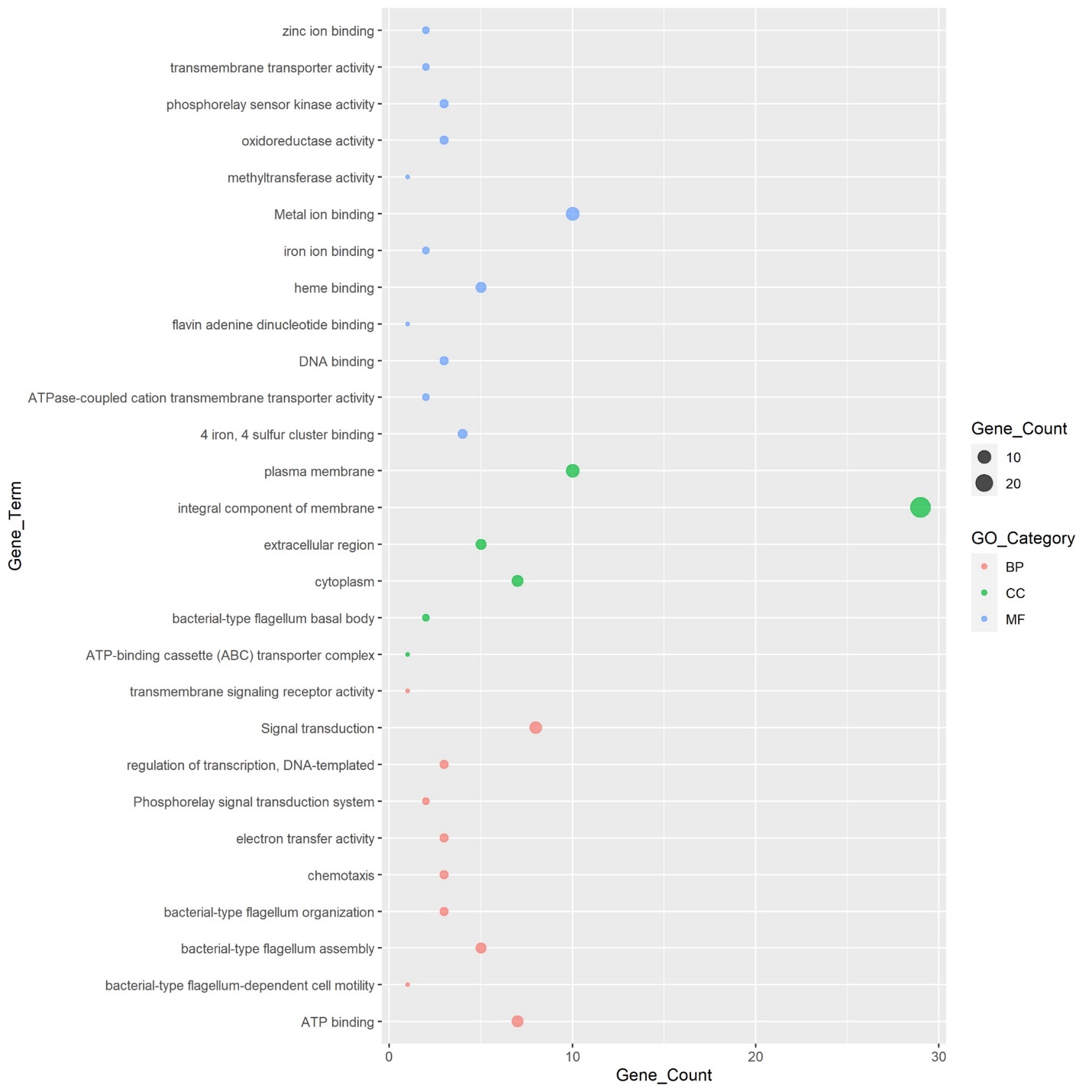
**Supplementary figure S7.** Gene Ontology bubble plot illustrating the enriched GO terms and their corresponding gene counts in *D. vulgaris* – control vs. 25 mg/L CuO (EC-10). BP - Biological process; CC - Cellular component; MF - Molecular function


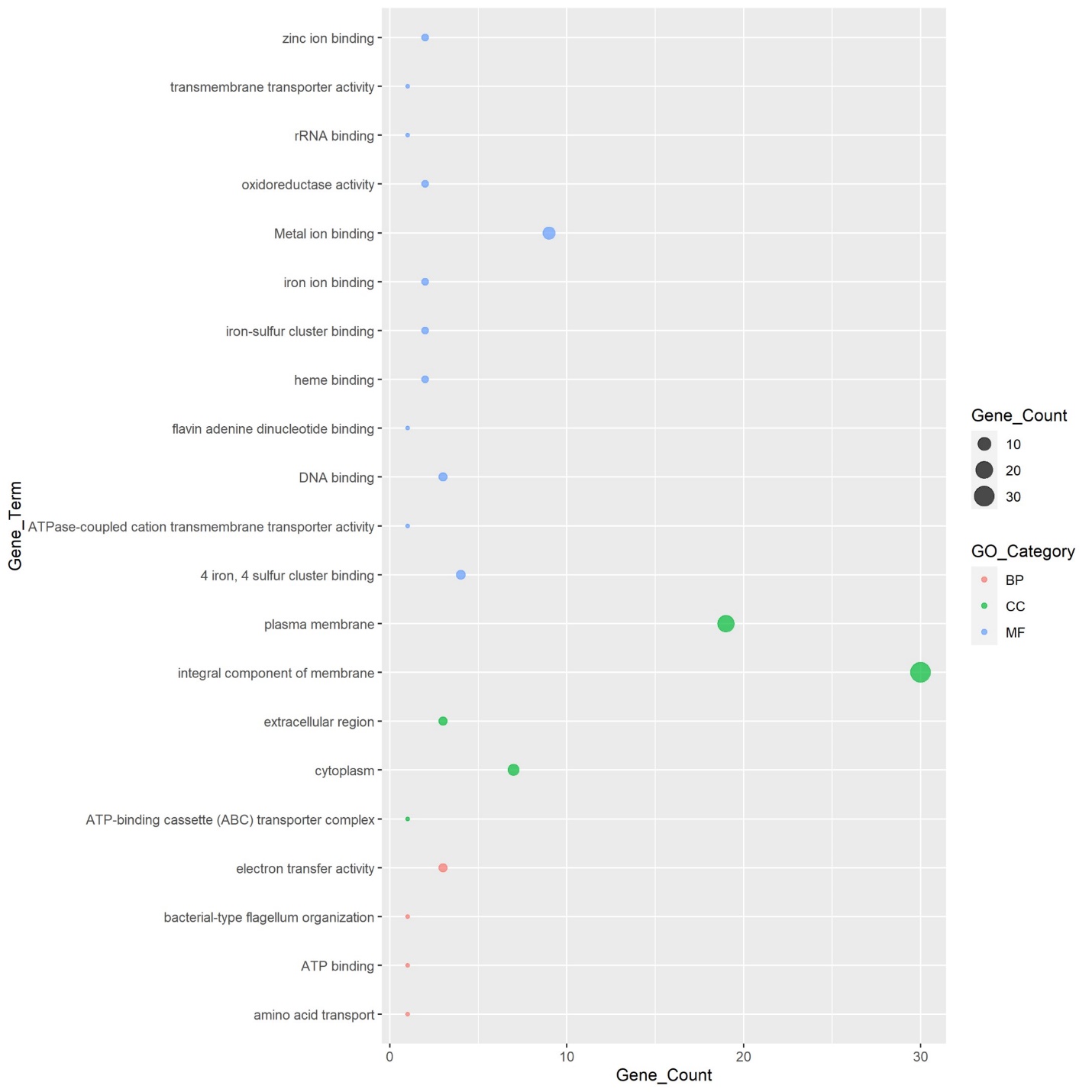


**Supplementary figure S8.** Gene Ontology bubble plot illustrating the enriched GO terms and their corresponding gene counts in *D. vulgaris* – 25 mg/L CuO vs. 250 mg/L CuO (EC-11). BP - Biological process; CC - Cellular component; MF - Molecular function


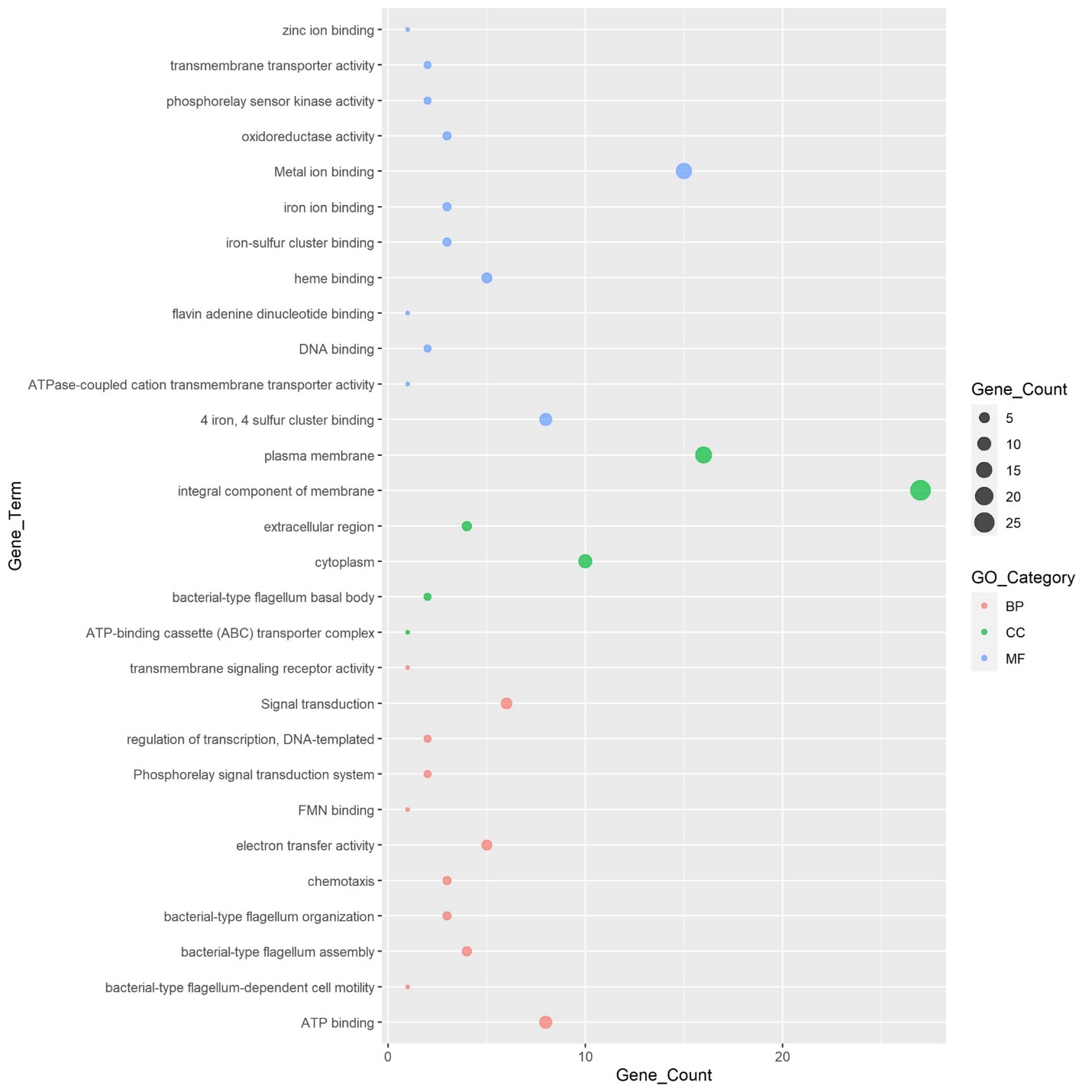


**Supplementary figure S9.** Gene Ontology bubble plot illustrating the enriched GO terms and their corresponding gene counts in *D. vulgaris* – 250 mg/L CuO vs. control (EC-12). BP - Biological process; CC - Cellular component; MF - Molecular function


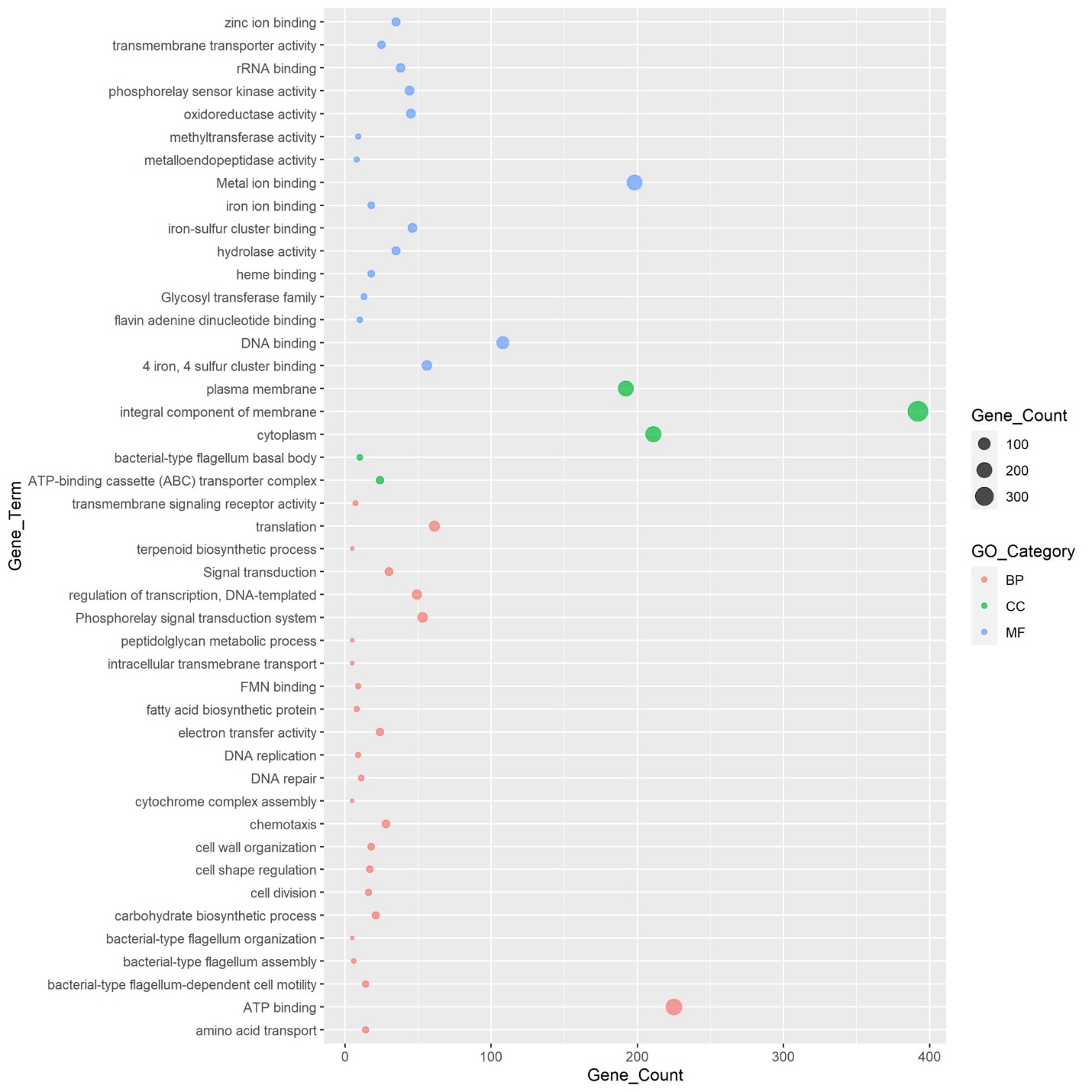


**Supplementary figure S10.** Gene Ontology bubble plot illustrating the enriched GO terms and their corresponding gene counts in *D. vulgaris* – control vs. 4 μg N/Liter FNA (EC-13). BP - Biological process; CC - Cellular component; MF - Molecular function

**Supplementary table. S1.** Up and down-regulated pathways related to electrochemical interactions and biofilm with z-scores that showed statistically significant DGE.

| Pathway | EC-1 | | EC-3 | |
| --- | --- | --- | --- | --- |
|  | **Genes involved** | **z-score** | **Genes involved** | **z-score** |
| Cell motility and flagella | Dde_2708, Dde_0385, Dde_0355, Dde_3159, Dde_3589, Dde_0353, Dde_3586, Dde_3155, Dde_3585, Dde_3158, Dde_3151, Dde_3156, Dde_0356 | -0.23 (13) | Dde_0356, Dde_3158, Dde_3156, Dde_3151, Dde_3155, Dde_3585, Dde_3586, Dde_0355, Dde_0353, Dde_3159, Dde_0354, Dde_0352, Dde_2708 | 0.23 (13) |
| Riboflavin biosynthesis/ FMN binding proteins | Dde_1104, Dde_1788, Dde_3141, Dde_3505, Dde_0187, Dde_0785, Dde_0441, Dde_0750, Dde_0039, Dde_3667, Dde_2434 | 0.06 (11) | Dde_3505, Dde_0187, Dde_1170, Dde_0441, Dde_0750, Dde_0039, Dde_3667, Dde_2435 | 0.05 (8) |
| Electron transfer activity | Dde_0813, Dde_0683, Dde_0561, Dde_0286, Dde_2954, Dde_3954, Dde_2749, Dde_2943, Dde_0291, Dde_3513, Dde_0586, Dde_0681, Dde_3266, Dde_0580, Dde_0717, Dde_0653, Dde_1256, Dde_0527, Dde_2460, Dde_0082, Dde_3667 | 0.11 (21) | Dde_0286, Dde_3266, Dde_0813, Dde_0291, Dde_2944, Dde_0561, Dde_2943, Dde_3513, Dde_0717, Dde_2460, Dde_3194, Dde_0427, Dde_0653, Dde_0082, Dde_1256, Dde_3667 | 0 (16) |
| Lactate oxidation | Dde_3245, Dde_1843 | -0.04 (2) | Dde_3245. Dde_1843 | 0.04 (2) |
| Chemotaxis and Two-component system | Dde_3585, 2040, Dde_3212, Dde_3586, Dde_2585, Dde_0575, Dde_0352, Dde_1571, Dde_3068, Dde_0385, Dde_1665, Dde_1077, Dde_2104, Dde_2708, Dde_1575, Dde_0391, Dde_2411, Dde_1196, Dde_0369, Dde_1078, Dde_3508 | 0.02 (21) | Dde_3585, Dde_3586, Dde_0575, Dde_0281, Dde_0352, Dde_2585, Dde_1627, Dde_2040, Dde_0703, Dde_1665, Dde_3100, Dde_2708, Dde_1077, Dde_1196, Dde_0369, Dde_0391, Dde_1571, Dde_2411, Dde_3508 | 0.02 (19) |
| Stress response genes | Dde_0882, Dde_3193, 2066, Dde_0713, Dde_1311, Dde_1027 | -0.04 (5) | Dde_3193, Dde_2066, Dde_0713, Dde_1027, Dde_2010 | 0.02 (5) |

**Supplementary table. S2.** The top six enriched GOs along with their GO IDs, total gene counts and z scores of thirteen experimental stress conditions of SRBs evaluated in this study.

| A | GO ID | GO terms | Total gene count |
| --- | --- | --- | --- |
|  | GO:0016021 | integral component of membrane | 414 |
|  | GO:0005524 | ATP binding | 220 |
|  | GO:0046872 | metal ion binding | 191 |
|  | GO:0005886 | plasma membrane | 187 |
|  | GO:0005737 | cytoplasm | 175 |
|  | GO:0003677 | DNA binding | 133 |
| B | GO ID | **GO terms** | **Total gene count** |
|  | GO:0016021 | integral component of membrane | 82 |
|  | GO:0005524 | ATP binding | 52 |
|  | GO:0046872 | metal ion binding | 45 |
|  | GO:0005886 | plasma membrane | 38 |
|  | GO:0005737 | cytoplasm | 36 |
|  | GO:0003677 | DNA binding | 32 |
| C | GO ID | **GO terms** | **Total gene count** |
|  | GO:0016021 | integral component of membrane | 321 |
|  | GO:0005524 | ATP binding | 189 |
|  | GO:0046872 | metal ion binding | 167 |
|  | GO:0005886 | plasma membrane | 154 |
|  | GO:0005737 | cytoplasm | 140 |
|  | GO:0003677 | DNA binding | 99 |
| D | GO ID | **GO terms** | **Total gene count** |
|  | GO:0016021 | integral component of membrane | 265 |
|  | GO:0005737 | cytoplasm | 134 |
|  | GO:0005524 | ATP binding | 133 |
|  | GO:0046872 | metal ion binding | 123 |
|  | GO:0005886 | plasma membrane | 119 |
|  | GO:0000160 | Phosphorelay signal transduction system | 48 |
|  | GO:0006412 | Translation | 48 |
| E | GO ID | **GO terms** | **Total gene count** |
|  | GO:0016021 | integral component of membrane | 173 |
|  | GO:0046872 | metal ion binding | 80 |
|  | GO:0005886 | plasma membrane | 79 |
|  | GO:0005737 | cytoplasm | 73 |
|  | GO:0005524 | ATP binding | 67 |
|  | GO:0000160 | Phosphorelay signal transduction system | 28 |
| F | GO ID | **GO terms** | **Total gene count** |
|  | GO:0016021 | integral component of membrane | 120 |
|  | GO:0005737 | cytoplasm | 59 |
|  | GO:0005886 | plasma membrane | 55 |
|  | GO:0046872 | metal ion binding | 52 |
|  | GO:0005524 | ATP binding | 38 |
|  | GO:0003677 | DNA binding | 23 |
| G | GO ID | **GO terms** | **Total gene count** |
|  | GO:0016021 | integral component of membrane | 21 |
|  | GO:0005524 | ATP binding | 7 |
|  | GO:0005886 | plasma membrane | 6 |
|  | GO:0046872 | metal ion binding | 6 |
|  | GO:0003677 | DNA binding | 6 |
|  | GO:0005576 | Extracellular region | 5 |
| H | GO ID | **GO terms** | **Total gene count** |
|  | GO:0016021 | integral component of membrane | 156 |
|  | GO:0005886 | plasma membrane | 76 |
|  | GO:0005737 | cytoplasm | 60 |
|  | GO:0006412 | Translation | 40 |
|  | GO:0005524 | ATP binding | 35 |
|  | GO:0046872 | metal ion binding | 35 |
|  | GO:0019843 | rRNA binding | 28 |
| I | GO ID | **GO terms** | **Total gene count** |
|  | GO:0016021 | integral component of membrane | 104 |
|  | GO:0005737 | cytoplasm | 59 |
|  | GO:0005886 | plasma membrane | 46 |
|  | GO:0046872 | metal ion binding | 45 |
|  | GO:0005524 | ATP binding | 44 |
|  | GO:0006412 | Translation | 41 |
| J | GO ID | **GO terms** | **Total gene count** |
|  | GO:0016021 | integral component of membrane | 29 |
|  | GO:0046872 | metal ion binding | 10 |
|  | GO:0005886 | plasma membrane | 10 |
|  | GO:0007165 | Signal transduction | 8 |
|  | GO:0005737 | cytoplasm | 7 |
|  | GO:0005524 | ATP binding | 7 |
| K | GO ID | **GO terms** | **Total gene count** |
|  | GO:0016021 | integral component of membrane | 30 |
|  | GO:0005886 | plasma membrane | 19 |
|  | GO:0046872 | metal ion binding | 9 |
|  | GO:0005737 | Cytoplasm | 7 |
|  | GO:0051539 | 4 iron, 4 sulfur cluster binding | 4 |
|  | GO:0003677 | DNA binding | 3 |
|  | GO:0005576 | Extracellular region | 3 |
|  | GO:0009055 | electron transfer activity | 3 |
| L | GO ID | **GO terms** | **Total gene count** |
|  | GO:0016021 | integral component of membrane | 27 |
|  | GO:0005886 | plasma membrane | 16 |
|  | GO:0046872 | metal ion binding | 15 |
|  | GO:0005737 | Cytoplasm | 10 |
|  | GO:0051539 | 4 iron, 4 sulfur cluster binding | 8 |
|  | GO:0005524 | ATP binding | 8 |
| M | GO ID | **GO terms** | **Total gene count** |
|  | GO:0016021 | integral component of membrane | 392 |
|  | GO:0005524 | ATP binding | 225 |
|  | GO:0005737 | Cytoplasm | 211 |
|  | GO:0046872 | metal ion binding | 198 |
|  | GO:0005886 | plasma membrane | 192 |
|  | GO:0003677 | DNA binding | 108 |

**Supplementary table S3.** Total number of protein-coding genes and families

| **Total # of genomes** | **Four**  **1. *O. alaskensis* G20**  **2. *D. vulgaris* Hildenborough**  **3. *D. hydrotehrmalis***  **4. *D. Piezophilus*** |
| --- | --- |
| **Total # of protein-coding genes** | **12821 genes with translation, 10178 are in homolog families, 2643 are in singleton families** |
| **Total # of families** | **5773 families 3130 homolog families 2643 singleton families** |

**Supplementary table S4.** The total number of homologous genes among the four SRBs evaluated in this study

| **Genome** | **Legend** | **G1** | **G2** | **G3** | **G4** |
| --- | --- | --- | --- | --- | --- |
| **G1** - *Oleidesulfovibrio alaskensis* G20 | # homolog families | 2546 | 1872 | 2143 | 1910 |
| **G2** - *Desulfovibrio hydrothermalis* AM13 = DSM 14728 | # homolog families | 1872 | 2303 | 1773 | 1981 |
| **G3** - *Desulfovibrio vulgaris* str. Hildenborough | # homolog families | 2143 | 1773 | 2399 | 1815 |
| **G4** - *Pseudodesulfovibrio piezophilus* C1TLV30 | # homolog families | 1910 | 1981 | 1815 | 2333 |
